# Naproxen and Ibuprofen Exposure Alter the Blood–Testis Barrier in a Novel In Vitro Model

**DOI:** 10.3390/ijms27073033

**Published:** 2026-03-26

**Authors:** Krista M. Symosko Crow, In Ki Cho, Robert Clayton Edenfield, Kristen F. Easley, Ana Planinić, Nagham Younis, Elizabeth Waters, James S. McClellan, Amanda Colvin Zielen, Kylie Tager, Carlos Castro, Calvin Simerly, Kyle E. Orwig, Davor Ježek, Michael Koval, Charles A. Easley

**Affiliations:** 1 Department of Environmental Health Science, College of Public Health, University of Georgia, Athens, GA 30602, USA; ksymosko18@gmail.com (K.M.S.C.); elizabeth.waters@uga.edu (E.W.);; 2 Regenerative Bioscience Center, University of Georgia, Athens, GA 30602, USA; 3 Division of Pulmonary, Allergy, Critical Care and Sleep Medicine, Department of Medicine, Emory University School of Medicine, Atlanta, GA 30322, USA; 4 Centre of Excellence for Reproductive and Regenerative Medicine, School of Medicine, University of Zagreb, 10000 Zagreb, Croatia; ana.planinic@mef.hr (A.P.); davor.jezek@mef.hr (D.J.); 5 Department of Obstetrics, Gynecology and Reproductive Sciences, Magee-Womens Research Institute, University of Pittsburgh Medical Center, Pittsburgh, PA 15213, USA; 6 Pittsburgh Development Center, Division of Developmental & Regenerative Medicine, University of Pittsburgh Cancer Institute, University of Pittsburgh Medical Center, Pittsburgh, PA 15213, USA; 7 Center for Cystic Fibrosis and Airways Disease Research, Emory University School of Medicine, Atlanta, GA 30322, USA; 8 Department of Cell Biology, Emory University School of Medicine, Atlanta, GA 30322, USA

**Keywords:** non-steroidal anti-inflammatory drugs (NSAIDs), naproxen, ibuprofen, Sertoli cells, blood–testis barrier (BTB), rhesus macaques, non-human primate, infertility

## Abstract

Semen parameters, including sperm counts, have rapidly declined in men across the globe over the last five decades. Although this decline remains unexplained, lifestyle factors may affect male fertility. Recently, several studies highlighted a potential link between non-steroidal anti-inflammatory drug (NSAID) usage, such as naproxen and ibuprofen, and declining male fertility. However, the mechanisms by which these common analgesics affect male fertility, including their effects on the blood–testis barrier (BTB), remain poorly characterized. Utilizing an in vitro rhesus macaque non-human primate (NHP) BTB model, we demonstrate that serum levels of naproxen and ibuprofen alter the function of BTB. Following short-term naproxen and ibuprofen treatment of NHP primary Sertoli cells, we show that these NSAIDs increase the transepithelial electrical resistance, indicating an overall strengthening of the Sertoli cell junctions. Furthermore, naproxen and ibuprofen treatment alter the expression of genes involved in maintaining the BTB. Specifically, the genes that were significantly expressed in response to ibuprofen exposure were enriched for human phenotypic abnormalities linked to male factor infertility. Together, these results suggest that short-term naproxen and ibuprofen treatment disrupt the function of the BTB by altering the integrity of the Sertoli cell junctions, proposing a potential role of NSAIDs in male factor infertility.

## 1. Introduction

Over the past several decades, rising rates of male reproductive disorders and infertility have raised concerns about an emerging global reproductive health crisis [1]. Since the 1960s, these trends have been closely linked to an increased incidence of testicular dysgenesis syndrome (TDS), which affects approximately 1–3% of newborn boys [2]. TDS encompasses a spectrum of developmental and reproductive abnormalities, including infertility, cryptorchidism, hypospadias, and low testosterone, and is further associated with germ cell neoplasia in situ (GCNIS) and testicular neoplasms [2]. Although genetic and epigenetic factors contribute to TDS, a leading hypothesis is that modern environmental exposures play a central role in the observed decline in male reproductive health [2,3]. In particular, exposures to environmental toxicants and commonly used medications may disrupt sperm production, resulting in temporary or persistent infertility and potentially contribute to the increasing prevalence of TDS [2,4].

Over-the-counter (OTC) analgesics have recently gained attention for their potential to disrupt male fertility and act as endocrine-disrupting compounds. Among these, non-steroidal anti-inflammatory drugs (NSAIDs), a class of cyclooxygenase (COX) inhibitors that includes naproxen sodium (hereafter referred to as naproxen) and ibuprofen, are among the most widely used and environmentally prevalent pharmaceuticals worldwide ([5], reviewed in [6]), raising concerns about their global impact on reproductive health [7]. Prenatal exposure is particularly concerning: male offspring of women who used NSAIDs during pregnancy exhibit increased risks of cryptorchidism, hypospadias, shorter anogenital distance, low birth weight, premature closure of the ductus arteriosus, and reduced renal function ([8,9,10,11], reviewed in [12,13]). Despite these risks, NSAIDs remain among the most commonly used medications globally due to their effectiveness and accessibility for self-treatment of pain, minor ailments, and fever, especially among reproductive-aged men [4,14].

Ibuprofen use has been linked to endocrine-disrupting effects, including impaired Leydig cell function and increased luteinizing hormone levels secondary to reduced testosterone production [15]. This condition, known as compensated hypogonadism, is associated with all-cause mortality and a wide range of reproductive symptoms [16,17]. In contrast, naproxen appears to have minimal effects on reproductive hormone levels but is frequently listed among medications associated with delayed or retrograde ejaculation [18].

Beyond hormonal effects, NSAID therapy has been shown to alter additional biological processes and induce cytotoxicity. These drugs interact with cellular membranes and modify their biomechanical properties [19], including those of the gastric mucosal barrier, contributing to gastrointestinal bleeding and peptic ulcers (reviewed in [20]). Such findings raise questions about whether NSAID therapy similarly affects the blood–testis barrier (BTB). Sertoli cells form the BTB—a dynamic structure essential for spermatogenesis (reviewed in [21]). Several studies linked ibuprofen use to Sertoli cell dysfunction [15,22]. For example, ibuprofen reduces the stimulatory action of follicle-stimulating hormone (FSH), which is required to maintain spermatogenesis, and decreases inhibin B (*INHBB*) gene expression, a key regulator of FSH secretion, in an ex vivo adult human testis model. Together, these findings suggest that NSAID therapy may impair Sertoli cell function and, in turn, compromise developing germ cells [23].

When cultured in vitro, Sertoli cells form an epithelial barrier that closely resembles the functional and structural properties of the in vivo BTB [24,25]. When seeded on an extracellular matrix, these cells develop functional tight junctions that create a selective permeability barrier between neighboring cells [24,26,27]. This model has been used to evaluate the toxicity of environmental toxicants, such as perfluorooctane sulfonate [28], demonstrating its utility in identifying how environmental exposures perturb Sertoli cell function and BTB integrity [29,30,31,32]. Although adult Sertoli cells were historically considered terminally differentiated, evidence now shows that they have context-dependent proliferative capacity. Adult Sertoli cells can re-enter the cell cycle in vitro in both mouse and human systems [33], can be induced to proliferate in vivo under specific hormonal/seasonal conditions [34], and a proliferative subpopulation has been reported in an adult niche near the rete testis transition region [35]. In this context, the Sertoli cell barrier model offers a valuable platform for assessing the effects of OTC analgesics beyond the hormonal changes observed in healthy adult men following NSAID use.

This study aimed to determine how short-term NSAID treatment affects BTB function and integrity in vitro using primary Sertoli cells. Because of the inherent challenges in assessing the reproductive toxicity potential of drugs in adult humans, we employed a complementary set of approaches using non-human primate (NHP) primary Sertoli cells, a translationally relevant model that addresses the limitations of human studies. We investigated whether naproxen or ibuprofen directly alters barrier integrity using [1] reproductive toxicity assays, [2] whole transcriptome mRNA-sequencing to complement our in vitro approach, and [3] immunocytochemistry and immunohistochemistry to determine the presence of the NSAID-inhibiting enzymes in our model. Here, we demonstrate that short-term NSAID exposure alters the expression of junctional proteins in Sertoli cells, revealing that commonly used analgesics may influence BTB function and male reproductive health.

## 2. Results

### 2.1. Short-Term NSAID Treatment Alters the Barrier Function Without Affecting the Viability of In Vitro Primary Sertoli Cells

Several animal studies have demonstrated the adverse effects of NSAIDs on the development and function of Leydig and germ cells. Still, fewer studies have examined their impact on the Sertoli cells (reviewed in [12]). To address this gap, we first assessed whether naproxen or ibuprofen affected Sertoli cell viability or mitochondrial membrane potential, and whether any effects were dose-dependent. The NHP Sertoli cells were treated with naproxen or ibuprofen at concentrations selected to approximate reported therapeutic plasma levels in humans at 4 μm, 40 μm, or 400 μm for naproxen and 1 μm, 10 μm, or 100 μm for ibuprofen [15,36,37]. These concentrations approximate therapeutic plasma levels observed with common adult oral naproxen dosing (e.g., ~500–1000 mg/day) [36] or ibuprofen dosing (200–400 mg/day) [37], noting that the circulating drug is highly protein-bound and free-drug levels are correspondingly lower. Neither naproxen nor ibuprofen exposure significantly increased apoptosis or induced mitochondrial dysfunction at the concentrations tested (Figure S1).

**Figure 1:**
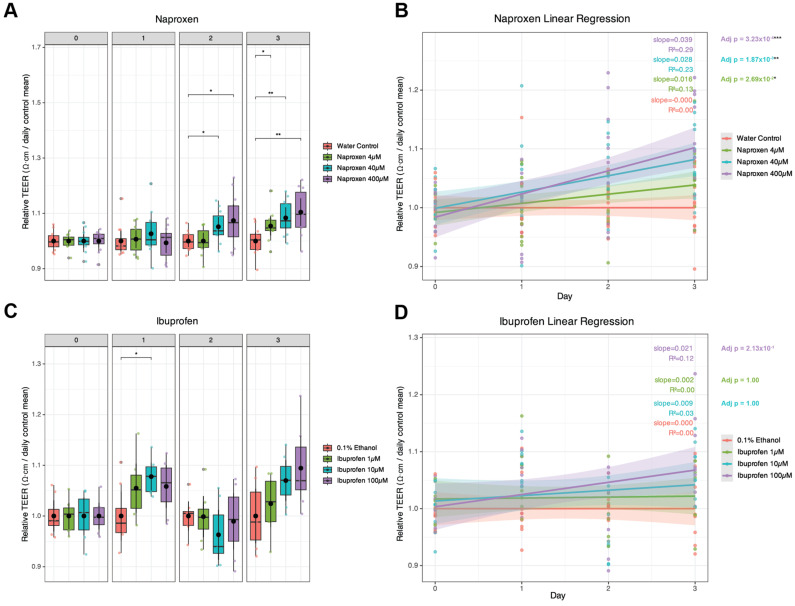
**Short-term NSAID treatment alters the Transepithelial Electrical Resistance (TEER) of non-human primate (NHP) Sertoli cells in vitro.** In order to assess the integrity of the blood–testis barrier and Sertoli cell tight junctions, TEER was measured. (**A**) Short-term treatment with naproxen and ibuprofen (**C**) serum levels significantly increased the TEER, reflecting a strengthening in the integrity of the Sertoli cell tight junctions. Twelve (*n* = 12) replicates for naproxen and eight (*n* = 8) replicates for ibuprofen were performed for each condition. TEER values were day-normalized to the daily control mean (water for naproxen; ethanol for ibuprofen). For each day, pairwise comparisons were performed between each concentration and its respective control using two-sample *t*-tests (control vs. each dose within the same day). Linear regression analysis (**B**,**D**) was performed using ordinary least squares regression (TEER = β0 + β1·Day); slope significance was evaluated by the two-sided *t*-test for β1 with Holm correction across conditions. *p*-values were Holm-adjusted within each day to correct for multiple dose comparisons, and significance was displayed on the plots as asterisks using * *p* < 0.05, ** *p* < 0.01, and *** *p* < 0.001.

Barrier function, assessed by measuring transepithelial electrical resistance (TEER), showed that both naproxen and ibuprofen increased TEER by Day 3 (Figure 1). Naproxen at 40 µM and 400 µM significantly increased TEER on Day 2 (*p* = 0.0128, *p*.adj = 0.0384 and *p* = 0.0203, *p*.adj = 0.0406), and a dose-dependent increase in TEER on Day 3 (*n* = 12; Figure 1A) compared to the water-only control. Linear regression of all three concentrations showed significance (*p*.adj = 0.0269 for 4 µM, *p*.adj = 0.00187 for 40 µM, and *p*.adj = 3.23^−04^ for 400 µM, Figure 1B). Treatment with 10 µM ibuprofen significantly increased TEER on Day 1 (*n* = 8; Figure 1C) compared to the 0.1% ethanol control. Although 100 µM ibuprofen also increased TEER on Day 1 (*p* = 0.05, *p*.adj = 0.1) and Day 3 (*p* = 0.0182, *p*.adj = 0.0546), these effects did not reach statistical significance. Linear regression analysis showed a general increase in the trend. Still, it did not achieve significance (*p*.adj = 0.21 for 100 µM ibuprofen, Figure 1D). Together, these results indicate that short-term naproxen and ibuprofen exposure increases TEER, suggesting a strengthening in the Sertoli cell barrier function. Since these data suggest that each drug alters Sertoli cell BTB integrity, we hypothesized that the functional changes in the in vitro BTB would be driven by altered expression of BTB-related genes.

### 2.2. Short-Term NSAID Treatment Alters the Expression of Genes Involved in the Function of the NHP Blood–Testis Barrier

**Figure 2:**
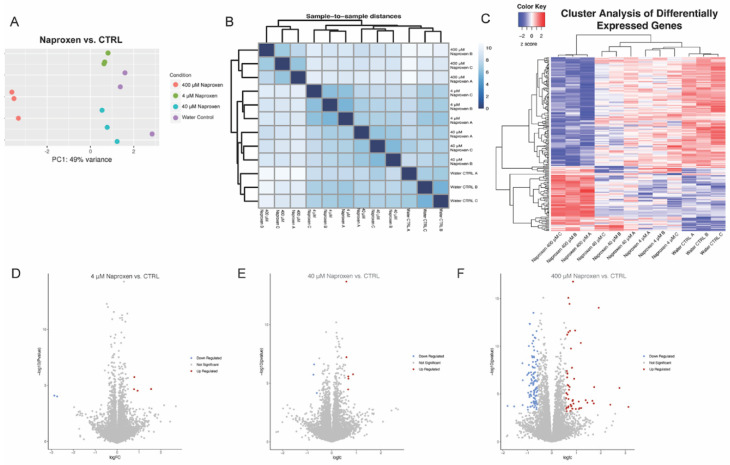
**Ribonucleic acid sequencing analysis of non-human primate (NHP) primary Sertoli cells exposed to naproxen in vitro.** (**A**) Principal component analysis plot of three biological replicates after exposure to 4 μm, 40 μm, and 400 μm naproxen compared to water vehicle control. (**B**) Poisson distance plot showing dissimilarities among the samples based on the transcriptome profiles after exposure to 4 μm, 40 μm, and 400 μm naproxen compared to the water vehicle control. (**C**) Hierarchical clustering of differentially expressed genes in NHP primary Sertoli cells exposed to 4 μm, 40 μm, and 400 μm naproxen compared to water vehicle control. (**D**–**F**) Volcano plots depicting −log_10_ range of differentially expressed genes that show ±1.5 fold change and *p*-adjusted values (*p*.adj; using Benjamini and Hochberg’s approach for controlling the false discovery rate) less than 0.01. (**D**) 6 genes in the 4 μm exposure sample, 4 genes up-regulated (red) and 2 genes down-regulated (blue), (**E**) 9 genes in the 40 μm exposure sample, 6 genes up-regulated (red) and 3 genes down-regulated (blue), and (**F**) 158 genes in the 400 μm exposure sample, 53 genes up-regulated (red) and 105 genes down-regulated (blue). The heatmap (**C**) shows normalized gene expression from limma, row-wise normalized.

To determine whether naproxen or ibuprofen alters the transcriptional landscape of Sertoli cells, we treated NHP primary Sertoli cells with plasma serum-relevant concentrations of each drug or vehicle controls for five days, isolated total RNA, and performed mRNA-sequencing (RNA-seq). Principal component analysis and Poisson distance plots revealed that NHP primary Sertoli cells exposed to 400 µM naproxen are distinctly different from the 4 µM and 40 µM naproxen groups and the water control (Figure 2A,B). The 400 µM naproxen dose showed the most significant transcriptional changes, with the greatest number of differentially expressed genes compared to the water control and the 4 µM and 40 µM naproxen doses (Figure 2C; Data files S1–S3). Compared to the control, 4 µM naproxen resulted in 6 significantly differentially expressed genes (4 up-regulated and 2 down-regulated; Figure 2D), 40 µM naproxen resulted in 9 significant differentially expressed genes (6 up-regulated and 3 down-regulated; Figure 2E), and 400 µM naproxen resulted in 142 significant differentially expressed genes (53 genes up-regulated and 105 genes down-regulated; Figure 2F), highlighting a concentration-dependent effect. At 4 µM and 40 µM, the gene expression profiles were similar to the water control (Figure 2C).

**Figure 3:**
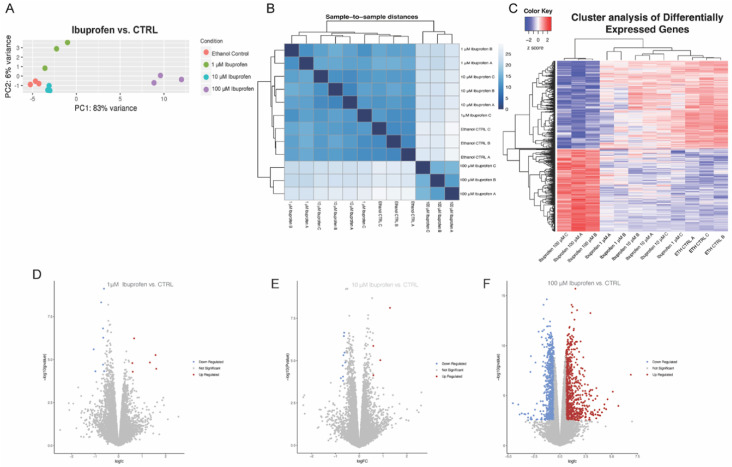
**Ribonucleic acid sequencing analysis of non-human primate (NHP) primary Sertoli cells exposed to ibuprofen in vitro.** (**A**) Principal component analysis plot of three biological replicates after exposure to 1 μm, 10 μm, and 100 μm naproxen compared to a water vehicle control. (**B**) Poisson distance plot showing dissimilarities among the samples based on the transcriptome profiles after exposure to 1 μm, 10 μm, and 100 μm ibuprofen compared to the ethanol vehicle control. (**C**) Hierarchical clustering of differentially expressed genes in NHP primary Sertoli cells exposed to 1 μm, 10 μm, and 100 μm ibuprofen compared to ethanol vehicle control. (**D**–**F**) Volcano plots depicting −log_10_ range of differentially expressed genes that show ±1.5 fold change and *p*-adjusted values (*p*.adj; using Benjamini and Hochberg’s approach for controlling the false discovery rate) less than 0.01. (**D**) 14 genes in the 1 μm exposure sample, 6 genes up-regulated (red) and 8 genes down-regulated (blue), (**E**) 13 genes in the 10 μm exposure sample, 4 genes up-regulated (red) and 9 genes down-regulated (blue), and (**F**) 1,567 genes in the 100 μm exposure sample, 801 genes up-regulated (red) and 766 genes down-regulated (blue). The heatmap (**C**) shows normalized gene expression from limma, row-normalized.

Ibuprofen had a similar pattern. Principal component and Poisson distance plot analyses showed that NHP primary Sertoli cells exposed to 100 µM ibuprofen were distinct from the 1 µM and 10 µM groups and the 0.1% ethanol control (Figure 3A,B). The 100 µM dose had the most dramatic gene expression alterations relative to the 0.1% ethanol control and 1 µM and 10 µM ibuprofen doses (Figure 3C; Data files S4–S6). Compared with the control, 1 µM ibuprofen resulted in 14 significant differentially expressed genes (6 genes up-regulated and 8 genes down-regulated; Figure 3D), 10 µM ibuprofen resulted in 13 significant differentially expressed genes (4 genes upregulated and 9 genes down-regulated; Figure 3E), and 100 µM ibuprofen had 1,567 significant differentially expressed genes (801 genes up-regulated and 766 genes down-regulated; Figure 3F). As with naproxen, the 1 µM and 10 µM ibuprofen groups exhibited gene expression profiles similar to the vehicle control (Figure 3C). Additionally, selected genes involved in Sertoli cell and BTB functions were validated by RT-qPCR (Figure S2).

**Figure 4:**
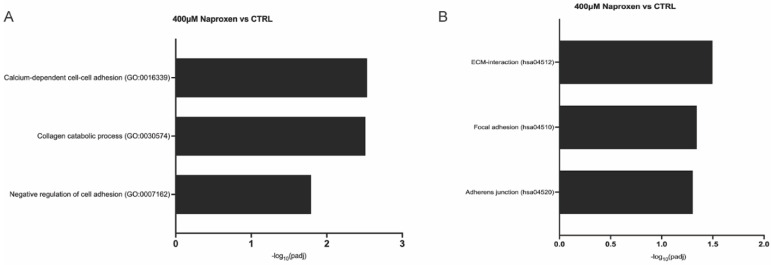
**Gene ontology (GO) annotation and Kyoto Encyclopedia of Genes and Genomes (KEGG) pathway enrichment analysis of differentially expressed genes in non-human primate (NHP) primary Sertoli cells exposed to 400 μm naproxen.** GO and KEGG pathway analysis of significantly enriched terms depicting −log_10_ range related to the blood–testis barrier (BTB) function, where the *p*.adj < 0.05 (*p*-values adjusted using the Benjamini and Hochberg’s approach for controlling the false discovery rate) in the (**A**) Biological processes and (**B**) KEGG pathways.

**Figure 5:**
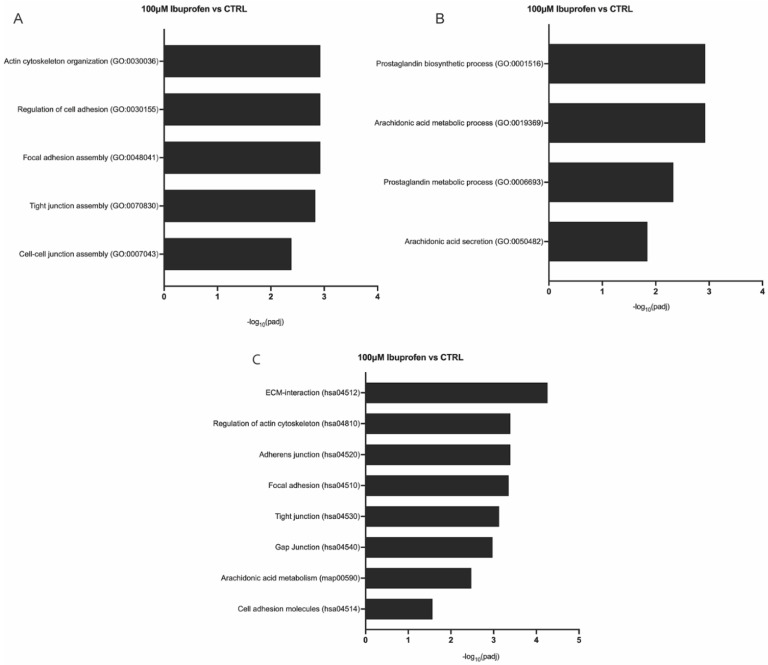
**Gene ontology (GO) annotation and Kyoto Encyclopedia of Genes and Genomes (KEGG) pathway enrichment analysis of differentially expressed genes in non-human primate (NHP) primary Sertoli cells exposed to 100 μm ibuprofen.** GO and KEGG pathway analysis of significantly enriched terms depicting −log_10_ range related to the blood–testis barrier (BTB) function and cyclooxygenase (COX) pathway, where the *p*.adj < 0.05 (*p*-values adjusted using the Benjamini and Hochberg’s approach for controlling the false discovery rate) in the (**A**) Biological processes enriched in BTB function. (**B**) Biological processes enriched in the COX pathway. (**C**) KEGG pathways enriched in BTB function and the COX pathway.

Gene Ontology (GO) and Kyoto Encyclopedia of Genes and Genomes (KEGG) pathway analyses revealed that both 400 µM naproxen and 100 µM ibuprofen altered pathways associated with BTB function and the cyclooxygenase pathway (Figure 4 and Figure 5). Differentially expressed genes were enriched in pathways essential for BTB and Sertoli cell function, including those involved in the cell cycle, cell division, DNA replication, translation, cell adhesion, androgen receptor signaling, and cell–cell signaling (Figure 4 and Figure 5; additional significantly enriched GO and Kegg analyses are shown in Data files S7–S10). At the 400 µM naproxen dose, GO and KEGG analyses identified significant enrichment of genes involved in calcium-dependent cell–cell adhesion (GO:0016339), collagen catabolic process (GO:0030574), negative regulation of cell adhesion (GO:0007162), ECM-interactions (hsa04512), focal adhesions (hsa04510), and adherens junctions (hsa04520) (Figure 4). Notably, cyclooxygenase-related pathways were not enriched following naproxen treatment. In contrast, 100 µM ibuprofen significantly enriched genes associated with actin cytoskeleton organization (GO:0030036), regulation of cell adhesion (GO:0030155), focal adhesion assembly (GO:0048041), tight junction assembly (GO:0070830), cell–cell junction assembly (GO:0007043), ECM-interactions (hsa04512), adherens junctions (hsa04520), gap junctions (hsa04540), prostaglandin biosynthetic (GO:0001516), arachidonic acid metabolic processes (GO:0019369), prostaglandin metabolic process (GO:0006693), and arachidonic acid secretion (GO:0050482) (Figure 5).

### 2.3. NSAID-Induced Gene Expression Changes Disrupt Blood–Testis Barrier Integrity by Affecting Junctional Dynamics

**Figure 6:**
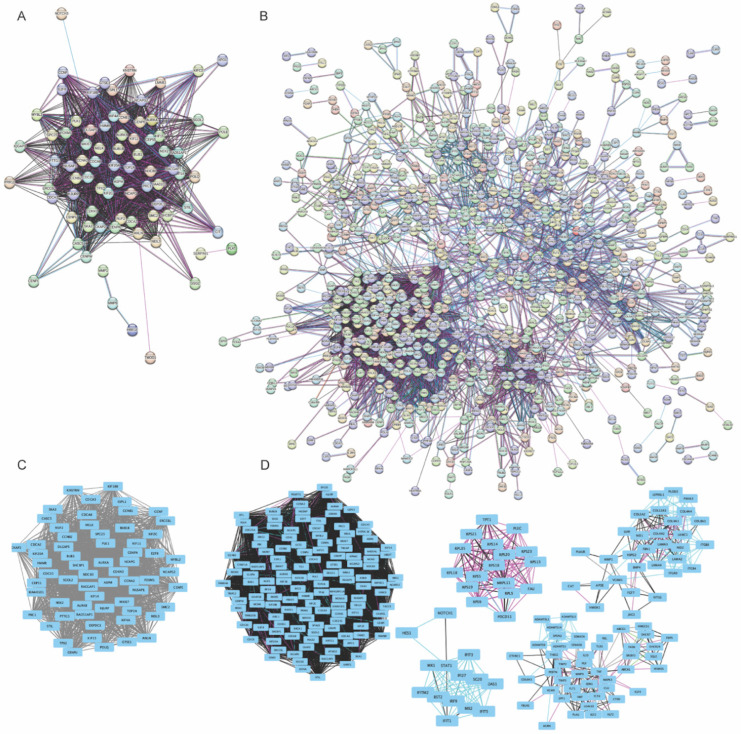
**Predicted protein–protein interaction (PPI) network and modules of differentially expressed genes from non-human primate (NHP) primary Sertoli cells exposed to naproxen and ibuprofen.** PPI networks of differentially expressed genes in Sertoli cells exposed to 400 μm naproxen or 100 μm ibuprofen were generated, with an interaction score of 0.400 as the cut-off criterion. (**A**) PPI network among genes for 400 μm naproxen. (**B**) PPI network among genes for 100 μm ibuprofen. (**C**) One module was identified from the naproxen genes with a score and number of nodes greater than 4. (**D**) Top 5 modules of the ibuprofen genes with a score and number of nodes greater than 4. Genes without identified interactions were excluded from the graphs. The colors of the edges represent the following: light blue = known interactions from curated databases, purple = known interactions that were experimentally determined, green = predicted interactions from gene neighborhood, red = predicted interactions from gene fusions, blue = predicted interactions from gene co-occurrence, black = co-expression, and light purple = protein homology.

The STRING database was used to construct protein–protein interaction (PPI) networks, which were then imported into Cytoscape (v.3.10.0) to explore the interactions among the differentially expressed genes. The MCODE plug-in (v.2.0.3) was applied for module analysis, and hub genes were identified using the CytoHubba software (v.0.1). For the 400 µM naproxen treatment, the resulting PPI network for the differentially expressed genes is shown in Figure 6A, with the corresponding module highlighted in Figure 6C. The significantly enriched functional module was associated with cell cycle, cell division, and DNA replication. The top three hub genes were *BUB1B*, *TOP2A*, and *KIF20A* (Data file S11). Notably, the network also included *CIT*, a key regulator of adherens junction integrity [38], which was downregulated following naproxen exposure (Figure 6A).

In contrast, STRING analysis of genes enriched in the primary Sertoli cells exposed to 100 µM ibuprofen revealed a more complex and interconnected PPI network (Figure 6B), with corresponding modules shown in Figure 6D. The most significantly enriched functional modules included pathways related to cell cycle, DNA replication, PI3K-Akt signaling, ECM-receptor interaction, and focal adhesion (Table 1). The top three hub genes identified were *CDK1*, *CCNB1*, and *GAPDH* (Data file S11). Importantly, ibuprofen exposure up-regulated several genes involved in maintaining BTB integrity, including *ADAMTS5, VCL*, and *MMP-9*. Both *ADAMTS5* and *VCL* are key regulators of adherens junction integrity [39], and *VCL* localizes to both Sertoli-Sertoli and Sertoli-Germ cell junctions [39]. These findings suggest that ibuprofen may disrupt the regulation of the adherens junction and potentially interfere with sperm release [40]. Together, these results indicate that although naproxen and ibuprofen act through distinct and multifaceted pathways, both drugs alter the expression of genes essential for BTB maintenance and junctional dynamics. The PPI-based findings are consistent with the paths identified through GO and KEGG analyses.

### 2.4. Human Phenotype Ontology Analysis Reveals Enrichment of Genes Associated with Male Infertility Following Ibuprofen Exposure

To further evaluate the potential reproductive consequences of NSAID-induced transcriptional changes, we performed human phenotype ontology (HPO) analysis on genes enriched in our primary NHP Sertoli cells treated with naproxen or ibuprofen.

Naproxen treatment was not associated with the selected male factor infertility phenotypes (Table 2). In contrast, ibuprofen exposure at 10 µM and 100 µM significantly enriched genes associated with diseases related to male factor infertility, including cryptorchidism (HP:0000028), functional abnormality of male internal genitalia (HP:0000025), aplasia/hypoplasia of the testes (HP:0010468), and infertility (HP:0000789) (Table 3). Collectively, the TEER measurements, gene set enrichment analyses, and STRING analyses indicate that NSAID exposure alters the expression of genes involved in Sertoli cell junctional function and BTB maintenance. These functional changes suggest a potential mechanistic link between NSAID therapy and compromised male fertility.

### 2.5. NHP Sertoli Cells and Human Testicular Tissue Express NSAID-Inhibiting Enzymes and Prostaglandins In Vitro and In Vivo

Naproxen and ibuprofen reduce pain and inflammation by inhibiting prostaglandin synthesis from arachidonic acid through the cyclooxygenase-1 (*PTGS1*/*COX1*) and cyclooxygenase-2 (*PTGS2*/*COX2*) enzymes [41,42,43]. Given our findings that NSAID exposure alters BTB function and junction-related gene expression, we next examined whether the COX/prostaglandin pathway is present in our in vitro NHP primary Sertoli cell model. RNA-seq analysis revealed that naproxen- and ibuprofen-exposed adult NHP primary Sertoli cells express the constitutive isoform, *PTGS1*, *PTGR1-3* (prostaglandin reductases 1-3), and multiple prostaglandin synthases and receptors, including *PTGDR* (which binds *PTGDS*), *PTGER2-4* (which binds *PTGES*), and *PTGIR* (which binds *PTGIS*) (Figure S3) [44,45,46,47]. Following 100 µM ibuprofen treatment, *PTGIS*, *PTGES*, *PTGDS*, and *PTGIR* mRNA levels significantly increased, while *PTGER4* and *PTGR2* mRNA significantly decreased. Notably, *PTGES* mRNA levels increased approximately 17-fold relative to the ethanol control, suggesting an ibuprofen-induced shift with 100 µM in prostaglandin biosynthesis that warrants further investigation. *PTGS2* mRNA was not detected in the NHP Sertoli cells under any treatment conditions.

**Figure 7:**
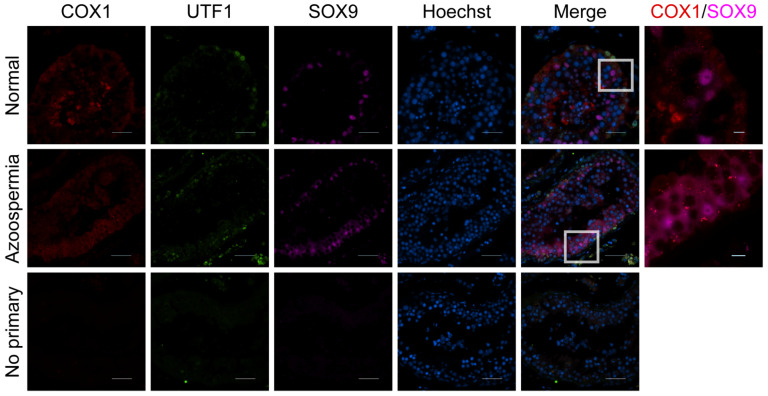
**Human testicular tissue expresses NSAID-inhibiting enzyme, COX1.** (**Left**) Representative immunofluorescent images of COX1 (red), UTF1 (green), SOX9 (purple), and Hoechst (blue) in a patient with full spermatogenesis (**top row**) and a patient with obstructive azoospermia with full spermatogenesis (**middle row**). (**Bottom**) Representative immunofluorescent secondary antibody only images where rbIgG (red), mIgG (green), gIgG (purple), and DAPI (blue). Scale bar 20 μm. (**Right**) Representative immunofluorescent staining of COX1 (red) and SOX9 purple in a patient with full spermatogenesis (**top**) and obstructive azoospermia with full spermatogenesis (**middle**). Scale bar 50 μm.

**Figure 8:**
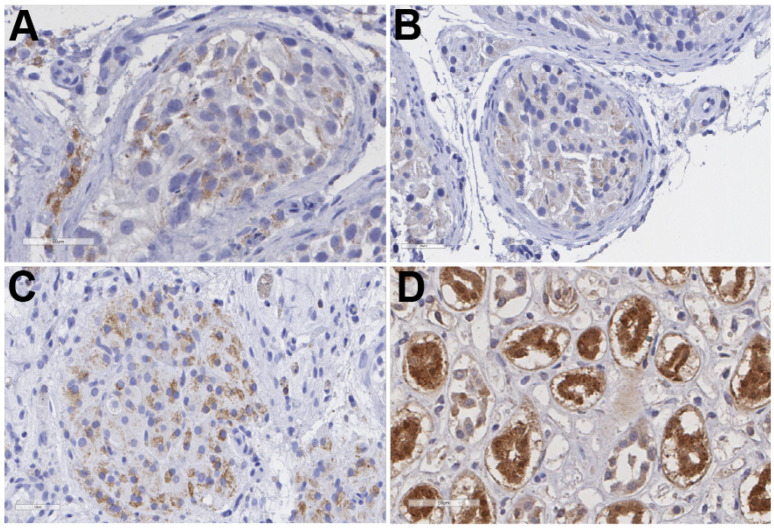
**Sertoli cells in human testicular tissue express the NSAID-inhibited enzyme, COX2.** Representative Hematoxylin and immunohistochemistry images of COX2 in human testicular biopsies from a male patient with (**A**) normal full spermatogenesis, (**B**) patient diagnosed with non-obstructive azoospermia with maturation arrest and mixed atrophy with inflammation, (**C**) patient who showed mixed atrophy, tubular fibrosis, and germ cell neoplasia in situ (GCNIS) with tumor, and (**D**) representative COX2 images from canine kidney as a positive control. Scale bar 50 μm.

To determine whether these pathways are also active in vivo, we examined COX expression in adult human testicular tissue. COX1 was detected within seminiferous tubules from testes with normal morphology by immunofluorescence (Figure 7), consistent with prior studies [48,49,50]. COX2 was detected in testicular biopsies from men with inflammation, impaired fertility (Figure 8B) [47,51,52], and testicular cancer (Figure 8C) [53], suggesting that diseased states within the testis can induce prostaglandin production. Both COX1 and COX2 were diffusely expressed throughout the seminiferous tubules, and COX1 colocalized with SOX9-positive Sertoli cells (Figure 7), suggesting that the COX/prostaglandin pathway may locally modulate Sertoli cell activity, BTB dynamics, and spermatogenesis [54] (Figure 7 and Figure 8).

## 3. Discussion

The BTB, formed by Sertoli cells, is essential for supporting spermatogenesis by regulating the entry of nutrients and signaling molecules, restricting toxicants from reaching the apical compartment where post-meiotic germ cell development occurs, and maintaining an immune-privileged environment within the seminiferous epithelium (reviewed in [55]). In the normal testis, the BTB divides the seminiferous epithelium into the basal compartment, containing spermatogonia and preleptotene spermatocytes, and the apical compartment, which includes pachytene spermatocytes and more mature spermatogenic cells [23]. Junctional complexes within the BTB allow the passing of small secondary messengers and mediate physical and physiological interactions between Sertoli cells and developing germ cells [56,57]. The BTB is composed of multiple coexisting junctional complexes, including tight junctions, gap junctions, desmosomes, and basal ectoplasmic specializations (including adherens junctions) [56], which together maintain compartmentalization within the seminiferous tubule and coordinate germ cell maturation in an immune-privileged state [23,55,58]. Unlike other epithelia, Sertoli cell tight junctions lie adjacent to the basement membrane, an altered form of the extracellular matrix (ECM) that plays a critical role in Sertoli cell tight junction barrier function, cell movement, and inflammatory responses [59,60]. ECM components (e.g., Type IV collagen and laminin), along with integrins, adapters (such as vinculin; *VCL*), and structural and signaling molecules, form an intricate network that maintains BTB architecture and function [60]. Perturbations to these structures, including those induced by pharmacological exposures, such as NSAIDs, have the potential to disrupt Sertoli cell function and impair spermatogenesis. Given the widespread use of OTC analgesics and emerging evidence of their unintended reproductive consequences in the human fetal [61,62,63,64,65] and adult testis [15,66], understanding their impact on the adult BTB is increasingly relevant for reproductive-aged men [67,68,69].

In this study, we demonstrate that short-term exposure to naproxen or ibuprofen does not affect the viability of primary NHP Sertoli cells but does alter BTB function and the transcriptional landscape of genes essential for junctional integrity. These findings are consistent with human fetal testis explant studies demonstrating that ibuprofen does not induce Sertoli cell death [22]. Importantly, increased TEER does not necessarily reflect improved barrier function. Because TEER primarily measures ionic resistance, it is sensitive to changes in tight junction protein composition or organization. The increases observed here are consistent with junctional tightening or reorganization rather than barrier leakiness or overt dysfunction. A leaky/compromised BTB disrupts the protected adluminal environment and is strongly associated with germ cell apoptosis, meiotic arrest, and infertility. However, spermatogenesis requires the BTB to be dynamic at specific stages; it must transiently restructure to allow preleptotene/leptotene spermatocytes to move from the basal to adluminal compartment while maintaining overall barrier integrity. If the barrier cannot remodel appropriately, germ cell transit and progression can be impaired.

This functional signature aligns with our RNA-seq findings, which reveal coordinated transcriptional changes in genes regulating adherens junctions after naproxen exposure and adherens, tight, and gap junctions after ibuprofen exposure. These results suggest that NSAID treatment alters BTB architecture through molecular remodeling. The TEER response to ibuprofen was non-monotonic, with the Day-1 pattern reappearing by Day-3. This suggests dynamic regulation of barrier properties rather than a simple linear dose effect and may reflect compensatory or adaptive epithelial responses (including transient partial recovery toward baseline at Day-2) and/or opposing concentration-dependent mechanisms, which may explain the lack of a significant linear dose trend at Day-2 and the re-emergence of a dose-related pattern by Day-3.

Because NSAID exposure in vivo can reduce testosterone production through effects on Leydig cell steroidogenesis and/or upstream HPG-axis regulation, we performed our experiments in a Sertoli cell-only culture system. We uniformly supplemented testosterone across all conditions to maintain a controlled androgen milieu and minimize phenotypic drift that can occur with androgen deprivation. This design was intentional: it allows assessment of the direct effects of NSAIDs on Sertoli cells while holding androgen signaling constant, rather than conflating NSAID activity with secondary consequences of altered testosterone availability.

Our RNA-seq analyses further revealed a dose-dependent response, with minimal gene expression changes at low NSAID concentrations and large coordinated shifts at high concentrations. This pattern suggests that at higher concentrations, naproxen and ibuprofen act on pathways beyond classical COX inhibition, engaging stress-response, cytoskeletal remodeling, and ECM-interaction pathways central to BTB dynamics. Enriched pathways identified through GO, KEGG, and STRING analyses, including cytoskeleton organization, focal adhesion, ECM–receptor interactions, and cell–cell junction assembly, are well-established regulators of BTB restructuring during spermatogenesis and homeostasis. Many of the differentially expressed genes, such as *VCL*, *ADAMTS5*, *CIT*, and *MMP-9*, are known regulators of Sertoli cell junctions, reinforcing the biological relevance of these findings.

A particularly interesting result from the GO and KEGG analyses was the enrichment of pathways related to tight junction barrier function. Previous studies have shown that BTB function can be disrupted by altered production of collagen, laminins, and matrix metalloprotease-9 (*MMP-9*), all of which are critical for maintaining the integrity of the BTB and Sertoli cell tight junctions [40,70,71]. Consistent with this, 400 µM naproxen significantly decreased *MMP-9*, and 100 µM ibuprofen significantly increased it (Data files S1 and S4). Furthermore, 100 µM ibuprofen significantly decreased the expression of several collagen and laminin genes (*COL12A1*, *COL1A1*, *COL4A1*, *COL4A2*, *COL5A2*, *COL6A3*, *LAMA2*, *LAMA3*, *LAMA4*, and *LAMC1*; Data file S4), suggesting that NSAID exposure may compromise in vitro BTB integrity through dysregulation of ECM components and junctional remodeling.

Ibuprofen produced a more pronounced transcriptional response than naproxen, consistent with its documented endocrine-disrupting potential, its known effects on Sertoli cell signaling (including FSH responsiveness and *INHBB* expression), and its greater environmental and physiological prevalence. These observations align with the existing literature and suggest that ibuprofen may exert broader or more potent effects on Sertoli cell biology than naproxen.

Importantly, our Human Phenotype Ontology (HPO) analysis further revealed that genes altered by ibuprofen were significantly enriched in phenotypes associated with male factor infertility, including cryptorchidism, testicular hypoplasia, and impaired spermatogenesis. These phenotypes mirror those reported in epidemiological studies of NSAID exposure and in fetal testis explant models and are mechanistically consistent with disrupted Sertoli cell function. The convergence of functional, transcriptional, and phenotype-level evidence strengthens the mechanistic link between NSAID-induced Sertoli cell dysfunction and male reproductive outcomes.

Although our in vitro system cannot fully replicate the longer dosing regimens experienced by many reproductive-aged men and elite athletes, it allowed us to model early events associated with repeated NSAID exposure. With the highest concentrations of naproxen (400 µM) and ibuprofen (100 µM) exceeding typical human plasma levels, they are useful for defining mechanistic thresholds in vitro. Because both drugs are highly protein-bound, the free drug concentration in our culture system is expected to differ from the nominal dose and will depend on serum content and lot composition. All treatment groups, including vehicle controls, were cultured in the same base medium with identical FBS concentration, minimizing between-group differences attributable to serum binding or serum-derived factors. NSAIDs can partition into lipid-rich tissues, including the testis, and local tissue exposure may differ from circulating levels due to protein binding, tissue distribution, and repeated dosing. High-dose in vitro exposures are commonly used to reveal early cellular stress responses, junctional remodeling programs, and cytoskeletal changes that may be subtle or undetectable at lower concentrations but could become relevant with chronic or high-frequency NSAID use. Accordingly, the dose-dependent transcriptional and functional changes observed here highlight molecular vulnerabilities in adult Sertoli cells and provide a rationale for follow-up in vivo studies to test whether similar pathways are engaged. The use of primary NHP Sertoli cells, free of germ cell contamination, provided a unique opportunity to interrogate adult Sertoli cell biology directly (Figure S5), laying the groundwork for future in vivo NHP studies to examine how chronic NSAID use influences spermatogenesis and fertility outcomes.

Because NSAIDs exert their therapeutic effects by inhibiting COX enzymes and prostaglandin synthesis [5,15,63,72], we also examined whether the COX/prostaglandin pathway is present and responsive in adult Sertoli cells. Prostaglandins play important roles in male reproductive function (reviewed in [54]), and recent single-cell RNA-seq data show expression of *PTGS1* and *PTGS2* within the Sertoli cell population (Figure S5 and Table S4). However, the specific functions of prostaglandins in adult Sertoli cells remain poorly defined, in part because most prior studies relied on immature rodents to avoid contamination from germ and other somatic cells. Our data show that NHP Sertoli cells express *PTGS1* and multiple prostaglandin synthases and receptors, and that ibuprofen significantly alters the expression of several prostaglandin-related genes, including a striking 17-fold increase in *PTGES*. Although species differences exist in COX and prostaglandin expression, our findings align with previous reports describing the presence of both COX isoenzymes, prostaglandins, and their receptors in Sertoli cells [44,45,46,47]. These data indicate that our in vitro NHP Sertoli cell model retains key components of the COX/prostaglandin pathway and can serve as a relevant system for future mechanistic studies.

Several limitations should be acknowledged. We did not directly assess protein-level changes in junctional components such as claudin-11, occludin, ZO-1, N-cadherin, or VCL. Although our RNA-seq and pathway analyses strongly implicate these proteins in NSAID-induced BTB remodeling, future studies incorporating immunoblotting, immunofluorescence, or super-resolution imaging will be essential to determine how transcriptional changes translate into structural alterations. Such protein-level validation will further clarify how NSAIDs influence the dynamic assembly and disassembly of Sertoli cell junctions.

Additionally, our functional assessment of the BTB was limited to TEER. While TEER captures key aspects of barrier physiology, it does not fully reflect the dynamic remodeling required for germ cell transit, nor does it assess actin bundle organization, Sertoli cell polarity, or endocytic recycling of junctional proteins. Future studies incorporating live-cell imaging, actin cytoskeleton analysis, and assays of BTB opening and resealing will be important for defining how NSAIDs influence the full spectrum of BTB dynamics.

Complementary immunofluorescence studies in human testicular tissue revealed COX1 expression in normal seminiferous tubules and COX2 expression in testes from men with inflammation, impaired fertility, or testicular cancer. The localization of COX1 to SOX9-positive Sertoli cells suggests that prostaglandin signaling may play a previously underappreciated role in BTB regulation and Sertoli cell function [54]. Together, these findings demonstrate that NHP Sertoli cells and human testicular tissue express key enzymes and receptors of the COX/prostaglandin pathway, and that this pathway is responsive to NSAID exposure. Given that Sertoli cells contribute to testicular immune privilege, NSAID-induced alterations in prostaglandin signaling could influence local immune responses, potentially contributing to inflammation or idiopathic infertility [73,74]. In such cases, macrophages and mast cells shift from the interstitial space to the seminiferous tubule compartment in the testes of men with infertility [75,76]. The absence of PTGS2 (COX2) expression in our NHP Sertoli cell cultures, despite its presence in human testicular biopsies and single-cell RNA-seq data, likely reflects the inducible nature of COX2, which is typically low or undetectable under basal conditions but is upregulated in response to inflammatory cues, cytokines, or cellular stress. Our culture conditions may not replicate the inflammatory microenvironment present in human testes, underscoring the importance of considering both basal and inducible prostaglandin pathways when evaluating NSAID effects on testicular physiology.

When integrated with our functional and transcriptomic results, these data support a model in which NSAIDs alter BTB integrity and Sertoli cell junctional dynamics, at least in part, through modulation of prostaglandin-related signaling. This framework informs the broader implications of NSAID use on male reproductive health and sets the stage for discussing their potential impact on male fertility. More broadly, our work identifies early molecular events through which commonly used analgesics can reshape adult Sertoli cell biology, revealing previously unrecognized vulnerabilities in the BTB. By establishing a mechanistic link between NSAID exposure, junctional remodeling, and pathways associated with male infertility, this study provides a foundation for future in vivo investigations and underscores the need to reevaluate how routine NSAID use may influence reproductive health in men. By identifying early molecular disruptions in Sertoli cell function, this work highlights the BTB as a potential target of routine NSAID use and emphasizes the importance of evaluating these common medications within the context of male fertility.

## 4. Materials and Methods

### 4.1. Study Design

This study aimed to uncover how naproxen and ibuprofen, two common OTCs, alter the function and integrity of the NHP Sertoli cell BTB in vitro. NHP Sertoli cells were isolated from the rhesus monkey testis tissue. Through serial subculture and morphological selection, a stable and highly homogeneous primary cell line was established, and the identity of cells was verified through RT-qPCR, ICC, and IHC, confirming the identity of Sertoli cells. We treated NHP Sertoli cells with plasma serum concentrations of naproxen and ibuprofen to determine cell viability, apoptosis, and mitochondrial membrane potential (triplicate), revealing that neither naproxen nor ibuprofen exposure significantly increased apoptosis or caused mitochondrial dysfunction in the primary NHP Sertoli cell culture. The impact of naproxen and ibuprofen treatment on the integrity of the BTB was assessed by performing TEER (*n* = 12 for naproxen; *n* = 8 for ibuprofen). Using RNA-seq (triplicate), we observed alterations in gene expression of BTB-related genes by ibuprofen and naproxen, indicating the functional changes from TEER resulted from the alterations in gene expression. As the primary enzyme in response to inflammation and the primary target for NSAIDS, we verified the presence of cyclooxygenases and prostaglandins in Sertoli cells, suggesting a role of the COX/ prostaglandin pathway in BTB homeostasis and Sertoli cell function. For each experiment, there were 3 replicates for each gene, and 4 plates per vehicle control and analgesic concentration, representing 4 separate and distinct biological replicates (*n* = 12) except for the Sertoli cell validation experiments (*n* = 3).

### 4.2. Primary Non-Human Primate Sertoli Cell Isolation and Validation

#### 4.2.1. Isolation

The isolation of Sertoli cells from adult rhesus macaque testicular tissue was adapted from previously described human testicular cell isolation methods [77]. Briefly, the Sertoli cells were isolated using a Percoll (Cat. 17089101, Cytiva, Marlborough, MA, USA) gradient and purified by visual analysis. For this study, the cells routinely contained greater than 90–95% primary Sertoli cells.

#### 4.2.2. Validation

The non-human primate (NHP) Sertoli cell cultures were validated by real-time quantitative polymerase chain reaction (RT-qPCR) and immunocytochemistry (ICC), as further described below. Briefly, the Sertoli cells were validated by various NHP Sertoli cell markers (*SOX9*, *AR*, *WT1*, and *GATA4)*, Fibroblast markers (*DPP4* and *FSP1*), Peritubular cell marker (*FN1*), Myoid marker (*ACTA2*), spermatogenic markers (*MAGEA4* and *TNP1*), and Leydig cell markers (*STAR* and *INSL3*) (Integrated DNA Technologies, Table S2) by RT-qPCR. Additionally, these cells were validated by immunocytochemistry analysis for *SOX9* and *WT1* (EMD Millipore, Abcam, Invitrogen, Table S2), described below.

### 4.3. Cell Culture and NSAID Treatment

#### 4.3.1. Cell Culture

The NHP primary Sertoli cells were cultured and maintained in DMEM 1X (Cat. 11965092, Gibco, Grand Island, NY, USA), supplemented with 10% Fetal Bovine Serum (Cat. 35-010-CV, Corning, Corning, NY, USA), 5% Penicillin-Streptomycin (Cat. No. 15142122, Gibco, USA), and 5% MEM Non-Essential Amino Acids Solution (Cat. No. 11140050, Gibco, USA) in a 5% CO_2_ and 95% humidified air incubator at 35 °C. Media was exchanged every 48 h and passaged at 80–90% confluency.

#### 4.3.2. NSAID Treatment

Cells were treated with naproxen (Cat. N8280, MilliporeSigma, Burlington, MA, USA) or ibuprofen (Cat. I14883, MilliporeSigma, USA) at concentrations selected to reflect reported plasma levels in healthy adult men (10^−4^–10^−5^ M) [15]. Confluent cultures received naproxen at 4 µM, 40 µM, or 400 µM, or ibuprofen at 1 µM, 10 µM, or 100 µM. Cells were maintained in DMEM containing 10% FBS, with naproxen dissolved in water and ibuprofen dissolved in 0.1% ethanol or water; matched vehicle-only controls were included. Media (including NSAID or vehicle) were replaced daily. The ethanol vehicle was used at minimal volume and is unlikely to cause cell death or appreciable effects.

For cell viability, apoptosis, and mitochondrial membrane potential assays, primary Sertoli cells were treated for three days in media containing an NSAID, as cultures reached ~95% confluency by Day 3. For TEER, RT-qPCR, and mRNA-sequencing analyses, we used a two-step exposure design to model short-term NSAID exposure in our in vitro BTB system while minimizing confounding effects of prolonged culture. Primary NHP Sertoli cells were expanded to confluency under standard conditions; however, because cultures reached maximal confluency and showed reduced viability by Day 5, pre-seeding exposure was limited to 24 h. Accordingly, confluent cells were pretreated for 24 h with naproxen, ibuprofen, or the corresponding vehicle control (identical handling and solvent concentration), then dissociated and seeded onto 12 mm Transwell inserts (Costar, Corning^®^ Life Sciences, Corning, NY, USA) coated with Matrigel (Corning^®^) at 1 × 10^6^ cells/cm^2^ to initiate barrier formation. Treatments were maintained in both apical and basolateral compartments after seeding to ensure barrier assembly occurred under defined exposure conditions while reducing stress associated with extended pre-seeding culture.

TEER measurements began the following day (Day 0; Figure 1A,C). After each daily measurement, cells received fresh NSAID or vehicle treatment along with 10 µM testosterone (Cat. No. T1500, Sigma, St. Louis, MO, USA) to stimulate Sertoli cell activity for an additional three days.

### 4.4. Cell Viability and Apoptosis

Cell viability and mitochondrial membrane potential were examined using previously established methods [78,79,80]. By utilizing the Muse^®^ Annexin V and Dead Cell Assay Kit (Cat. No. MCH100105, Luminex, Austin, TX, USA), cell viability was assessed by measuring the percentage of apoptotic cells in the cultures by staining unfixed cells with Annexin V and 7-AAD as per the manufacturer’s instructions in preparation for flow cytometry. Each sample was analyzed on the Muse^®^ benchtop flow cytometer (MilliporeSigma, USA) and analyzed at 1000 events for three replications (*n* = 3) per analgesic concentration and water- or ethanol-only control.

### 4.5. Mitochondrial Membrane Potential

The mitochondrial membrane potential was assessed using the Muse^®^ MitoPotential Kit (Cat. No. MCH100110, Luminex, USA) to stain unfixed cells with the supplied dye and 7-AAD per the manufacturer’s instructions to prepare the samples for flow cytometry. Each sample was analyzed on the Muse^®^ benchtop flow cytometer (MillieporeSigma, Burlington, MA, USA) and analyzed at 1000 events for three replications (*n* = 3) per analgesic concentration and water- or ethanol-only control.

### 4.6. Transepithelial Electrical Resistance (TEER) Measurement

The functionality of the BTB was assessed by performing TEER. Confluent cultures were treated with naproxen or ibuprofen for 24 h and then trypsinized and seeded at 1 × 10^6^ cells/cm^2^ in the apical (upper) chamber of the 12 mm diameter Transwell (Costar) coated with Matrigel^®^ (Corning^®^), as described above. The next day (Day 0), the TEER of the cells in NSAID-containing media was measured using an Epithelial Voltohmmeter (EVOM, World Precision Instruments, Inc., Sarasota, FL, USA) in ohms (Ω) × cm^2^, as previously described [81,82]. After the measurement, the cells were treated with ibuprofen, naproxen, or vehicle control, and 10 μm testosterone (Sigma). The TEER measurements were recorded, and media changes occurred for three consecutive days.

### 4.7. RNA Extraction and Quantification

RNA was extracted following the manufacturer’s instructions from the naproxen-, ibuprofen-, ethanol-only-, or water-only- treated NHP Sertoli cells using the RNeasy Plus Mini Kit (Qiagen, Germantown, MD, USA) after the TEER was measured on the third day to yield four biological replicates (*n* = 4). Using the NanoDrop^®^ 2000c spectrophotometer (Thermo Scientific, Waltham, MA, USA), RNA integrity was measured, and only 260/280 ratios > 2.0 were accepted for further processing. The samples were stored at −80 °C until RT-qPCR analysis and mRNA sequencing.

### 4.8. Reverse Transcription Real-Time Quantitative Polymerase Chain Reaction (RT-qPCR) Analysis

Each RNA sample (500 ng) was reverse transcribed to cDNA for RT-qPCR using the iScript™ cDNA Synthesis kit (Cat. No. 1708890, Bio-Rad, Hercules, CA, USA) as per the manufacturer’s instructions. Quantitative PCR was performed with 500 µg cDNA and the iQÔ SYBR Green Supermix (Cat. No. 1708880, Bio-Rad, USA) as per manufacturer’s instructions using the CFX Connect Real-Time PCR Detection System (Bio-Rad). The amplification parameters were as follows: the initial denaturation of 3 min at 95 °C, 40 cycles of 15 s denaturation at 95 °C, and 1 min at 57 °C for annealing and extension. After the final PCR cycle, a melt curve analysis was performed at the following parameters: 5 s per step, 65 °C to 95 °C at 0.5 °C increments.

All PCR primers (Integrated DNA Technologies, Table S1) were designed and validated for specificity and amplification efficiency to human genes *PTGS1*, *DPP4*, *FSP1*, *FN1*, and NHP genes *PTGS2*, *AR*, *SOX9*, *GATA4*, *CLDN3*, *CLDN4*, *CLDN8*, *CLDN11, ACTA2*, *STAR*, *INSL3*, *WT1*, *MAGEA4*, and *TNP1*. *GAPDH* was used as an internal control for normalization. For each experiment, there were 3 replicates for each gene, 4 plates were run per vehicle control, and analgesic concentration representing 4 separate and distinct biological replicates (*n* = 12) except for the validation experiments where 3 replicates (*n* = 3) were run for genes *DPP4*, *ACTA2*, *FSP1*, *FN1*, *STAR*, *INSL3*, *WT1*, *MAGEA4*, and *TNP1* (Figure S5A).

### 4.9. Immunocytochemistry (ICC)

NHP primary Sertoli cells cultured in the conditions described above were fixed in 4% paraformaldehyde (Cat. No. 15710, Electron Microscopy Solutions, Hatfield, PA, USA) for 15 min, then blocked with buffer containing 1× Phosphate-buffered Saline solution (PBS, Cat. No. 10010023, Gibco, USA), 0.1% Triton X (Cat. No. X100, Sigma, USA), and 5% normal goat serum (Cat. No. 092939149, MP Biomedicals, Santa Ana, CA, USA) or normal donkey serum (Cat. No. 017-000-121, Jackson ImmunoResearch Laboratories, West Grove, PA, USA) overnight at 4 °C as described [83]. For primary antibodies, *SOX9* and *WT1* (Table S2 and Figure S5B), incubation was performed overnight at 4 °C in blocking buffer, followed by three washes in 1X PBS with 0.1% Triton X for 10 min each at room temperature (RT) on an LSE Orbital Shaker (Corning^®^). Secondary antibody incubation (1:1000, Alexa Fluor™ Invitrogen, Carlsbad, CA, USA, Table S2) in blocking buffer occurred for 45 min at room temperature on the Orbital Shaker or overnight at 4 °C. Following the three washes described above, the samples were co-stained with Hoechst 33342 (1:1000, Cat. No. H1399, Invitrogen, Carlsbad, CA, USA).

### 4.10. Human Testis Samples Preparation and Histological Processing

#### 4.10.1. Participants

Sixteen patients with azoospermia were referred to an andrologist for male infertility at the Clinic of Urology at the University of Zagreb School of Medicine and were included in this study. Each patient was subjected to an open biopsy of the testis [84,85], as detailed below.

#### 4.10.2. Testicular Biopsy

The testicular biopsies were performed under spinal anesthesia, and a bilateral biopsy was performed when possible. After visualization of the testis and epididymis, a small surgical incision about 8–10 mm in length in the tunica albuginea of the testis was made, as described [86]. The testicular tissue was dissected using surgical microscissors, and five testicular samples were collected from different parts of the testis to be used for histological analysis, potential sperm extraction, and research purposes. According to Johnson’s score, the histological analysis identified one patient (*n* = 1; age 34 years) with fully preserved spermatogenesis [87]. Based on their clinical presentation and histological analysis, this patient was diagnosed with obstructive azoospermia. The remaining patients (*n* = 12) were diagnosed with various degrees of damage to the testicular parenchyma, including hypospermatogenesis, spermatogenic (maturation) arrest at either the spermatocyte or spermatid stages, Sertoli cell-only phenotype (syndrome), tubular fibrosis, and a combination of the previous testicular disorders known as mixed atrophy [87,88]. These patients were diagnosed with non-obstructive azoospermia. The average age of this group of patients was 39 years, with an age range of 33–47 years. Three patients (*n* = 3) were diagnosed with germ cell neoplasia in situ (GCNIS), a precursor to testicular germ cell tumors [89]. The average age of these patients was 39 years, with an age range of 34–45 years (Table S3).

#### 4.10.3. Tissue Processing

Briefly, testicular tissue was fixed in 10% neutral buffered formalin for 24 h and rinsed in water overnight. The tissue samples were then dehydrated in a series of increasing ethanol solutions. Following two xylene immersions for clearing, four paraffin immersions were performed. The infiltrated tissue was removed from the cassette and oriented within a metal mold. Then, the mold was filled with molten paraffin and transferred to the cold plate to set.

### 4.11. Immunofluorescence (IF) of Cyclooxygenase and Sertoli Cell Markers

Before proceeding with IHC, paraffin sections of 5 µM -fixed testicular biopsies were deparaffinized, cleared in xylene, and rehydrated in a graded ethanol series ending in PBS. Following high-temperature TRIS buffer antigen retrieval, the slides were blocked with 5% donkey (Jackson ImmunoResearch) or goat serum (MP Biomedical) for 60 min at room temperature (RT), after which they were incubated for 90 min at RT or overnight at 4 °C with primary antibody COX-1, UTF1, and SOX9 (Abcam, Invitrogen, Millipore/FisherSci, R&D Systems, antibody details in Table S2) diluted in blocking buffer containing 1× phosphate-buffered saline solution (Fisher Scientific, Hampton, NH, USA), 3% bovine serum albumin (BSA), and 0.1% Triton X-100 (Sigma) (3% BSA/PBS/0.1% Triton X-100). The slides were then rinsed with PBST (Tween-20, Cat. No. BP337-100, Fisher BioReagents, USA) (PBS/0.1% Tween-20), after which the primary antibodies were detected with secondary antibodies (Invitrogen, 1:200, Table S2) in blocking buffer for 45 min at RT. After the slides were washed in PBST three times at 5 min each, the samples were mounted with VectaShield mounting media containing DAPI (Cat. No. H-1200-10, Vector Laboratories, Inc., Burlingame, CA, USA). The appropriate positive and negative controls for each ICC and IF antibody were used. Images were processed using the NIS-Elements AR software (v.5.21.03 64-bit), with 3D deconvolution using the Richardson-Lucy deconvolution technique [90,91], followed by the creation of an extended depth of focus (EDF) document.

Following high-temperature Citrate buffer antigen retrieval, tissue sections were exposed to 3% hydrogen peroxide for 5 min. Samples were then blocked in Universal Blocking Reagent (Cat. No. HK085-5K, BioGenex, Fremont, CA, USA) for 5 min at RT. The samples were incubated for 60 min with an Anti-COX2 antibody (BD Transduction Laboratories™) in Renaissance Background Reducing Diluent (Cat. No. PD905, Biocare, Pacheco, CA, USA). Biotinylated horse anti-mouse secondary antibody (Cat. No. BA-2000, Vector Laboratories) was incubated on the tissue sections for 10 min at RT. The samples were labeled with 4plus Streptavidin HRP (Cat. No. HP604, Biocare Medical, Pacheco, CA, USA) and then submerged in Betazoid DAB Solution (Cat. No. BDB2004, Biocare Medical, USA) for 12 min. The samples were then counterstained with hematoxylin, followed by a bluing reagent stain (Cat. No. CS702-1G, Fisher, Waltham, MA, USA) in preparation for imaging. Except for the antigen retrieval and counterstain steps, all other steps of the COX-2 staining were carried out on the intelliPATH™ (Cat. No. IPS001, Biocare Medical, USA).

### 4.12. mRNA Sequencing (RNA-Seq), Quantification of Gene Expression Level, and Differential Gene Expression Analysis

NHP Sertoli cell culture, naproxen and ibuprofen exposure, and RNA extraction were carried out as previously described to yield three biological replicates (n = 3). Following extraction, the RNA samples were sent to Azenta Life Sciences (Azenta US, Inc., Burlington, MA, USA) for mRNA-sequencing (RNA-seq) analyses.

#### 4.12.1. Library Preparation and Illumina Sequencing

Azenta Life Sciences checks RNA quantity and quality in two ways: Qubit™ 2.0 Fluorometer (Invitrogen) and Agilent TapeStation (Agilent Technologies, Santa Clara, CA, USA). All mRNA samples sent to Azenta for sequencing passed their quality control. The workstation is described as follows: The RNA sequencing libraries were prepared using the NEBNext Ultra RNA Library Prep Kit for Illumina (Cat. No. E7530L, NEB, Ipswich, MA, USA) following the manufacturer’s instructions. Briefly, the mRNAs were enriched using Oligod(T) beads and were then fragmented for 15 min at 94 °C. Then, the first and second strand cDNA was subsequently synthesized. The cDNA fragments were end-repaired, and the 3′ ends of the DNA fragments were adenylated. The cDNA fragments were ligated to universal adapters, followed by index addition. The library was then generated by PCR amplification with limited cycles. The sequencing library was validated on the Agilent TapeStation (Agilent Technologies) and quantified using the Qubit™ 2.0 Fluorometer (Invitrogen) and quantitative PCR (KAPA Biosystems). The libraries were clustered and sequenced on an Illumina HiSeq instrument with paired-end 150 bp (PE150) sequencing. As described below, the initial data analysis, quality control, adapter sequence trimming, and alignment to the reference genome were performed at Azenta. From the Illumina instrument, the raw data files were transformed into raw sequenced reads by the Control base-calling software (RTA; v1.18.54). The raw sequence data were converted into FASTQ files and demultiplexed using Illumina’s bcl2fastq (v.2.17) software. Poor-quality adaptors and nucleotides were filtered out to leave clean reads using the Trimmomatic (v.0.36) [92] software. The clean paired-end reads were aligned to the reference genome (*Macaca mulatta*, build Mmul_10, downloaded from the ENSEMBL genome website browser) using the STAR aligner (v.2.5.2b) to generate BAM files.

#### 4.12.2. Quantification of Gene Expression Level and Differential Expression Analysis

The unique gene hit counts were calculated by using featureCounts from the Subread package (v.1.5.2). Transcripts Per Kilobase Million (TPM) was used to estimate the gene expression levels and then used for the downstream differential expression analysis. On the Galaxy server (usegalaxy.org), differential expression analysis for each group of samples (three distinct biological replicates) exposed to naproxen or ibuprofen compared to the control samples, water, and 0.1% ethanol, respectively, was performed using the limma-voom (Galaxy v.3.50.1 + galaxy0) method [93,94]. Genes were considered to have very low expression if they were below a minimum of 0.5 count-per-million (CPM) and were then filtered out if they did not meet this minimum CPM in at least two samples [95,96]. The resulting *p*-values were adjusted using the Benjamini–Hochberg method to control for the false discovery rate (FDR). This study used the trimmed mean of M-values (TMM) from the edgeR package (v.3.36.0) as the normalization method. Genes were considered differentially expressed with an adjusted *p*-value < 0.01 and 1.5-fold change in either direction. The differential expression analysis data and the normalized read counts were used to generate graphs on the Galaxy server using the Volcano Plot (Galaxy v.0.0.5) and heatmap2 (Galaxy v.3.1.1 + galaxy1) packages.

#### 4.12.3. Gene Set Enrichment Analysis

The Ensemble of Gene Set Enrichment Analysis (EGSEA; v.1.26.0) package was used to explore the biological signaling pathways and human phenotype ontology in the naproxen-treated samples compared to the water vehicle control and the ibuprofen-treated samples compared to the 0.1% ethanol vehicle control. The rhesus macaque gene symbols from limma-voom were matched to the *Homo sapiens* hg19 reference genome, downloaded from the Ensembl genome website browser for enrichment analysis. The Molecular Signatures Database [97] (MSigDB; v.2022.1) and Human Phenotype Ontology [98] (HPO; v.2022.1) collections were used to explore the phenotypic abnormalities in the human diseases collection. The terms from the human phenotypes associated with male factor infertility were used to assess further gene function, which was obtained from the NCBI Gene database (https://www.ncbi.nlm.nih.gov/gene), and biological relevance [99]. Enrichment results were considered significant if the adjusted *p*-value (FDR) was <0.05. The Gene Set Enrichment Analysis was performed in RStudio (v.2022.12.00) running on R Package (v.R-4.2.2).

### 4.13. Generation and Comprehensive Analysis of Protein–Protein Interaction (PPI) Network

We used the STRING database (https://string-db.org/) [100] to explore the relationship between DEGs following exposure to 400 µM naproxen or 100 µM ibuprofen. An interaction score of 0.400 (medium confidence) was used as the cutoff criterion, and the PPI network was visualized using STRING. The PPI networks were then imported into the Cytoscape software (v.3.10.0) for visual analysis. Then, the modules of the PPI network were screened using the MCODE (v.2.0.3) plug-in with a score and number of nodes greater than >4 to identify targets in the network. The top three hub genes were identified by the CytoHubba plug-in (v.0.1).

### 4.14. Statistical Analysis

GraphPad Prism software (v.9) and R Studio (version 2025.05.1 + 513) were used to analyze the cell viability, mitochondrial membrane potential, TEER, and RT-qPCR data. Significant differences in samples in comparison to the water-only control for naproxen and the ethanol-only control for ibuprofen were determined by the following methods: the cell viability and mitochondrial membrane potential were determined by a two-tailed, unpaired *t*-test, where * is *p* < 0.05, ** is *p* < 0.01, and *** is *p* < 0.001. For box-and-whisker plots, TEER values were day-normalized to the daily control mean (water for naproxen; ethanol for ibuprofen). For each day, pairwise comparisons were performed between each concentration and its respective control using two-sample *t*-tests (control vs. each dose within the same day). *p*-values were Holm-adjusted within each day to correct for multiple dose comparisons, and significance was displayed on the plots as asterisks using the same thresholds (*p*.adj < 0.05, ** <0.01, *** <0.001; ns is hidden). In the boxplots, the box represents the interquartile range (IQR), the center line indicates the median, and whiskers extend to 1.5 × IQR; individual replicate values are overlaid as points. In addition, the mean ± 95% confidence interval (CI) is shown for each group/day as a point with error bars. Linear regression was used to test for a time-dependent trend in TEER within each treatment condition. For each drug and each concentration (including the control), TEER values were first normalized within each day to the daily control mean (naproxen normalized to water control, such that the control mean equals 1.0 for each day and ibuprofen normalized to 0.1% ethanol). A separate ordinary least squares (OLS) linear model was then fit for each condition: TEER^day-norm^ =β_0_ + β_1_ (Day) + ϵ, where Day was treated as a numeric variable (Day 0–3). The slope (β1) represents the estimated change in normalized TEER per day. Statistical significance of the time trend was determined using the two-sided t-test for the slope coefficient (H0: β1 = 0). Model fit was summarized using R^2^. To account for multiple slope tests performed across concentrations within each drug, Holm’s multiple-comparison adjustment was applied to the slope *p*-values, and adjusted *p*-values were reported. Significance thresholds were defined as: *p*.adj < 0.05 (*), <0.01 (**), and <0.001 (***); otherwise not significant (ns).

The results from the RT-qPCR experiments were calculated by the ΔΔCT method as normalized fold differences in each target gene except for the NHP Sertoli cell validation experiments, where the relative fold change was calculated by the ΔCT method.

## 5. Conclusions

Despite the limited number of studies examining the reproductive consequences of NSAID use in adult men [15,101,102,103,104,105], the increasing global consumption of naproxen and ibuprofen underscores the need to better understand their long-term effects on male fertility [14,106,107,108]. Our findings add to a growing body of evidence that NSAIDs can influence male reproductive hormones, BTB function, and sperm production. As such, clinicians evaluating male infertility and counseling patients on family planning may need to consider OTC medication use as a potentially modifiable factor.

Together, our findings identify adult Sertoli cells as direct—and previously underrecognized—targets of NSAID action, demonstrating that short-term exposure to naproxen and ibuprofen alters BTB function and the transcriptional programs that maintain junctional integrity. Using an integrated functional-to-molecular approach with validation in human testis tissue, we link NSAID exposure to disrupted Sertoli cell pathways and potential male fertility risk. Dose-dependent changes in junctional, cytoskeletal, and prostaglandin-related signaling—particularly with ibuprofen—underscore the BTB’s sensitivity to commonly used analgesics and challenge assumptions about their reproductive safety. Given rising global NSAID use, these findings support incorporating OTC medication history into infertility evaluations and motivate in vivo studies to define how chronic exposure influences spermatogenesis, testicular health, and reproductive outcomes.

## Figures and Tables

**Table 1:** Module analysis of the top seven pathways in the protein–protein interaction (PPI) network of non-human primate (NHP) primary Sertoli cells treated with 100 μm ibuprofen.

Pathway Description	FDR Value	Number of Nodes	Genes
Cell cycle	2.91 × 10^−7^	33	*CDC6, GADD45B, MCM5, CDC25B, CCNB1, ESPL1, MCM4,* *PKMYT1, CDKN2C, E2F3, MCM6, CDK6, CCNA2, BUB1B,* *CCNB2, PLK1, BUB1, CDC25, AMCM7, PRKDC, CDC25C,* *SMC1A, ANAPC1, ATR, E2F1, E2F2, TTK, ORC1, CDC20, CDK1, CHEK1, CCNE2, MCM3*
DNA replication	8.80 × 10^−5^	15	*MCM5*, *POLE2*, *MCM4*, *LIG1, MCM6, POLA2, MCM7, POLE,* *PRIM1, RPA2, POLA1, RFC3, RFC4, RFC5, MCM3*
PI3K-Akt signaling pathway	2.10 × 10^−4^	52	*ITGB4, EPOR, COMP, ITGB8, COL1A1, KITLG, LAMA4, GNB4, LAMC1, VWF, GNB5, FLT4, PHLPP1, MAPK3, ITGA9, TNC, CDK6, FGF7, OSMR, FGF18, NTRK2, FLT1, PKN3, TGFA, COL6A3, MAP2K1, INSR, RPTOR, DDIT4, VEGFB, PPP2R1B, LAMA3, CREB3L2, ATF4, COL4A2, THBS2, EFNA1, EFNA3, COL4A1, PIK3CD, SPP1, COL4A4, IGF2, LAMA2, BRCA1, CCNE2, NR4A1, PGF, ITGB3, PHLPP2, VEGFA, GHR*
ECM-receptor interaction	2.10 × 10^−4^	22	*ITGB4, COMP, ITGB8, COL1A1, LAMA4, LAMC1, VWF, ITGA9,* *TNC, COL6A3, LAMA3, COL4A2, THBS2, HSPG2, COL4A1,* *AGRN, HMMR, SPP1, COL4A4, NPNT, LAMA2, ITGB3*
MicroRNAs in cancer	2.10 × 10^−4^	31	*HMOX1, CDC25B, KIF23, E2F3, MAPK3, NOTCH3, HDAC4, TNC, CDK6, TIMP3, CDCA5, NOTCH1, MAP2K1, CDC25A, RPTOR, DDIT4, CDC25C, E2F1, DNMT1, E2F2, EFNA1, EFNA3, MMP9, PLAU, IRS2, PIK3CD, BRCA1, CCNE2, DICER1, ITGB3, VEGF*
Human papillomavirus infection	2.90 × 10^−4^	48	*ITGB4, COMP, ITGB8, COL1A1, LAMA4, HES1, JAG1,* *LAMC1, WNT10A, VWF, MPP5, MAPK3, NOTCH3, WNT5A,* *ITGA9, TNC, CDK6, CCNA2, NOTCH1, WNT7A, BAX, COL6A3,* *TLR3, MAP2K1, PTGER4, PPP2R1B, LAMA3, CREB3L2, MX2,* *TRADD, ATR, E2F1, COL4A2, STAT1, THBS2, HEYL, COL4A1,* *PIK3CD, SPP1, COL4A4, IRF9, MX1, TNF, LAMA2, CCNE2, FZD4, ITGB3, VEGFA*
Focal adhesion	8.80 × 10^−4^	33	*ITGB4, VCL, COMP, ITGB8, COL1A1, LAMA4, LAMC1, VWF,* *FLT4, MAPK3, ITGA9, TNC, PAK1, FLT1, COL6A3, MAP2K1,* *VEGFB, TLN1, LAMA3, FLNC, MYLK, COL4A2, THBS2, FLNA,* *COL4A1, PIK3CD, SPP1, COL4A4, LAMA2, FLNB, PGF, ITGB3,* *VEGFA*

**Table 2:** Naproxen-induced non-human primate (NHP) primary Sertoli cell gene enrichment using the human phenotype ontology (HPO) database.

	4 μm Naproxen	40 μm Naproxen	400 μm Naproxen
Phenotype	*p*.adj	*p*.adj	*p*.adj
Cryptorchidism (HP:0000028)	0.08052002	0.08159682	0.12648019
Functional abnormality of male internal genitalia (HP:0000025)	0.08052002	1	0.85441472
Aplasia/ Hypoplasia of the testes (HP:0010468)	0.11567548	0.85137133	0.85664023
Infertility (HP:0000789)	0.28610804	1	0.97361985

Genes enriched in the diseases associated with male factor infertility are shown above. Diseases with a *p*-adjusted value (*p*.adj) < 0.05 were considered significant.

**Table 3:** Ibuprofen-induced non-human primate (NHP) primary Sertoli cell gene enrichment using the human phenotype ontology (HPO) database.

	1 μm Ibuprofen	10 μm Ibuprofen	100 μm Ibuprofen
Phenotype	*p*.adj	*p*.adj	*p*.adj
Cryptorchidism (HP:0000028)	0.15176734	0.00083239	8.9895 × 10^−8^
Functional abnormality of male internal genitalia (HP:0000025)	0.09505467	0.00103354	0.00054086
Aplasia/ Hypoplasia of the testes (HP:0010468)	0.10999084	0.0010258	0.00058149
Infertility (HP:0000789)	0.53214939	0.00094366	0.00320424

Genes enriched in diseases associated with male factor infertility are listed above. Diseases with a *p*-adjusted value (*p*.adj) < 0.05 were considered significant.

## Data Availability

The raw and processed RNA-seq data (Figure 2, Figure 3, Figure 4, Figure 5 and Figure 6, Figure S3; Table 1, Table 2 and Table 3, Data files S1–S11) underlying this article are available in NCBI SRA BioProject database at https://dataview.ncbi.nlm.nih.gov/object/PRJNA1241271?reviewer=u23r3p79beqd87vm116gk6lkk7 (release date 31 May 2026) and can be accessed with ID: PRJNA1241271. All values for all data points in graphs are reported in the Supporting Data Values file. All other data underlying this article are available in the article and in its online Supplementary Materials. The raw data will be shared based on reasonable requests to the corresponding author.

## Supplementary Materials

The following supporting information can be downloaded at: https://www.mdpi.com/article/10.3390/ijms27073033/s1. References [109,110] are cited in the supplementary materials.
