# Supplementary material for: Naproxen and Ibuprofen Exposure Alter the Blood–Testis Barrier in a Novel In Vitro Model
Source: Int J Mol Sci. 2026 Mar 26;27(7):3033. doi: 10.3390/ijms27073033 (PMC13072845; doi:10.3390/ijms27073033)
Supplement: Supplementary file 1 [file ijms-27-03033-s001.zip › 20260226_Revised Supplemental Figures.pdf]

Supplemental Figures: Ibuprofen and Naproxen Exposure alter the Blood-testis Barrier in a Novel in vitro model

Figure S1. Short-term NSAID treatment does not induce apoptosis or alter the mitochondrial membrane potential of non-human primate (NHP) primary Sertoli cells in vitro.

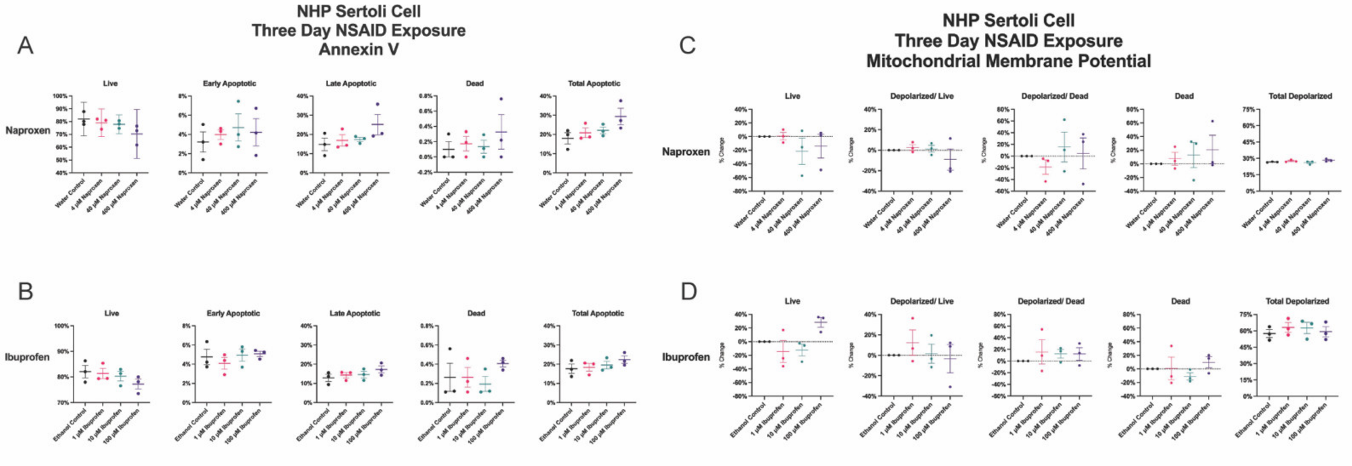

Short-term NSAID treatment does not alter the viability or mitochondrial membrane potential of NHP Sertoli cells. The Sertoli cells were treated for three consecutive days with 4 µM, 40 µM, and 400 µM naproxen and 1 µM, 10 µM, and 100 µM ibuprofen prior to the Annexin V (**A-B**) and mitochondrial membrane potential assays (**C-D**). (**A-B**) Graphical presentation showing that exposure to naproxen (**A**) or ibuprofen (**B**) does not induce apoptosis in the NHP Sertoli cells. (**C-D**) Graphical representation showing that exposure to naproxen (**C**) or ibuprofen (**D**) does not significantly decrease the mitochondrial membrane potential in the NHP Sertoli cells. A total of 1,000 events were acquired, with three replications ($n = 3$) performed for each drug treatment per assay. Significant alterations in cell viability and mitochondrial membrane potential were determined by a two-tailed Student's t-test, where * is $p < 0.05$, ** is $p < 0.01$, and *** is $p < 0.001$. Each bar represents the mean ± SEM.

Supplemental Figures: Ibuprofen and Naproxen Exposure alter the Blood-testis Barrier in a Novel in vitro model

Figure S2. Validation of Ribonucleic acid sequencing (RNA-seq) data by RT-qPCR.

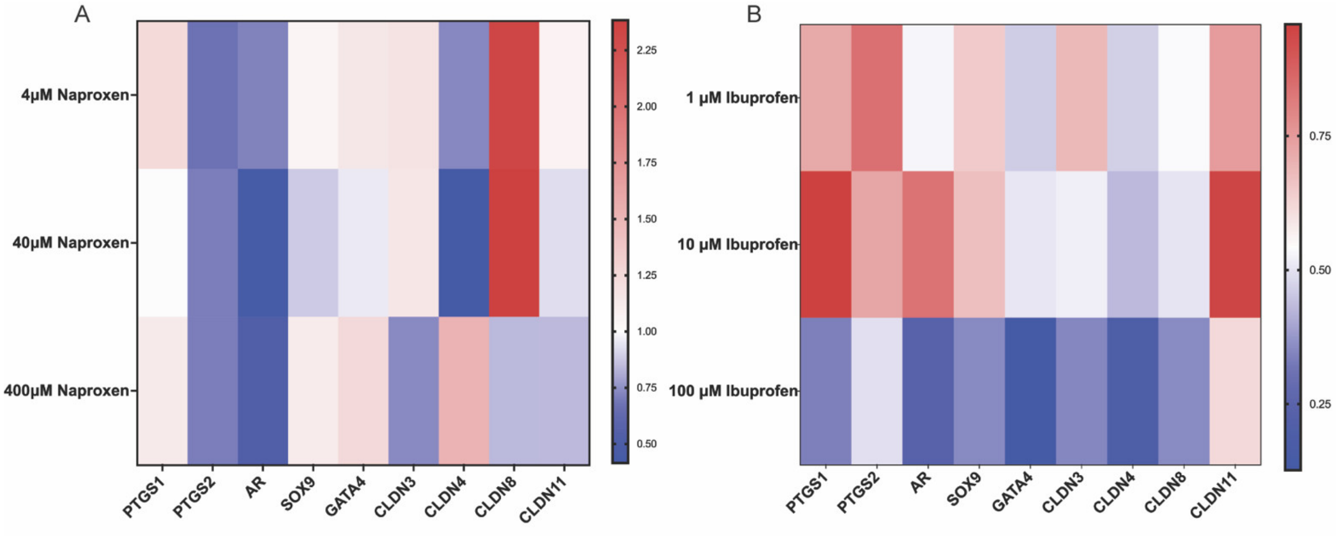

The results of the RT-qPCR validation of genes involved in Sertoli cell function (Androgen Receptor: *AR*, *SOX9*, and *GATA4*), tight junctions (*CLDN 3*, *4*, *8*, and *11*), and cyclooxygenases (*PTGS1* and *PTGS2)* from non-human primate primary Sertoli cells exposed to (**A**) 4 µM, 40 µM, and 400 µM naproxen compared to the water vehicle control (**B**) or 1 µM, 10 µM, and 100 µM ibuprofen compared to the ethanol control, compared to the normalized RNA-seq results in Figure 7. Each cell represents the mean of the normalized expression compared to the vehicle control from four (n = 4) replicates per drug per concentration (ΔΔCT).

Supplemental Figures: Ibuprofen and Naproxen Exposure alter the Blood-testis Barrier in a Novel in vitro model

Figure S3. Ribonucleic acid sequencing (RNA-seq) results of cyclooxygenases and prostaglandin expression in (NHP) primary Sertoli cells exposed to naproxen and ibuprofen in vitro.

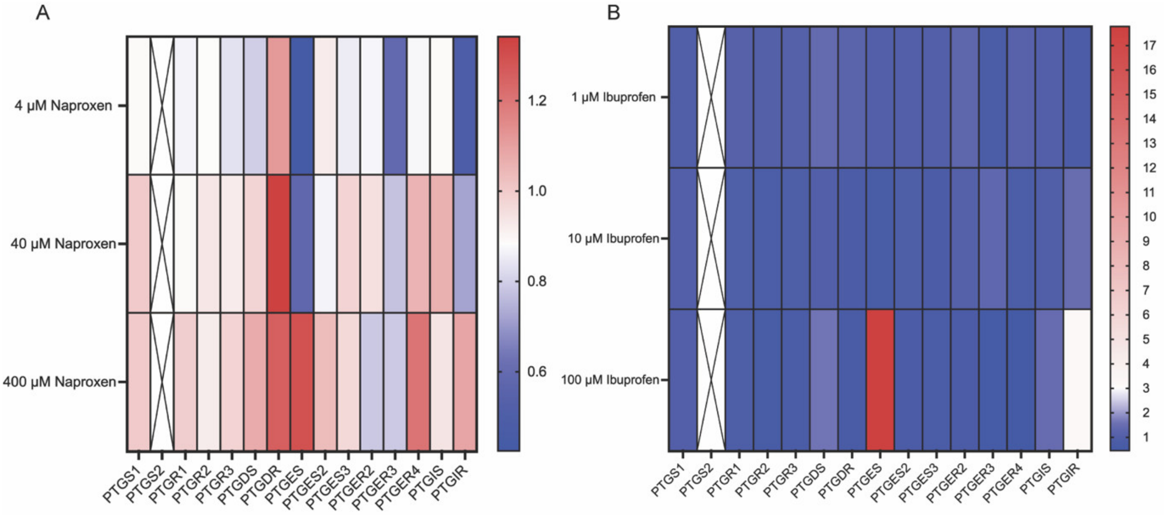

Heatmap visualization of RNA-seq gene expression analysis of genes involved in the cyclooxygenase pathway in NHP primary Sertoli cells exposed to (A) 4 µM, 40 µM, and 400 µM naproxen compared to water vehicle control or to (B) 1 µM, 10 µM, and 100 µM ibuprofen compared to the ethanol vehicle control. Each well represents the filtered counts from the limma, normalized to the vehicle control from three ($n = 3$) replicates per drug per concentration. The color intensity indicates expression levels, and white rectangles with an 'X' = not detected.

Figure S4. Non-human primate (NHP) primary Sertoli cells validated in vitro using qPCR and fluorescence microscopy.

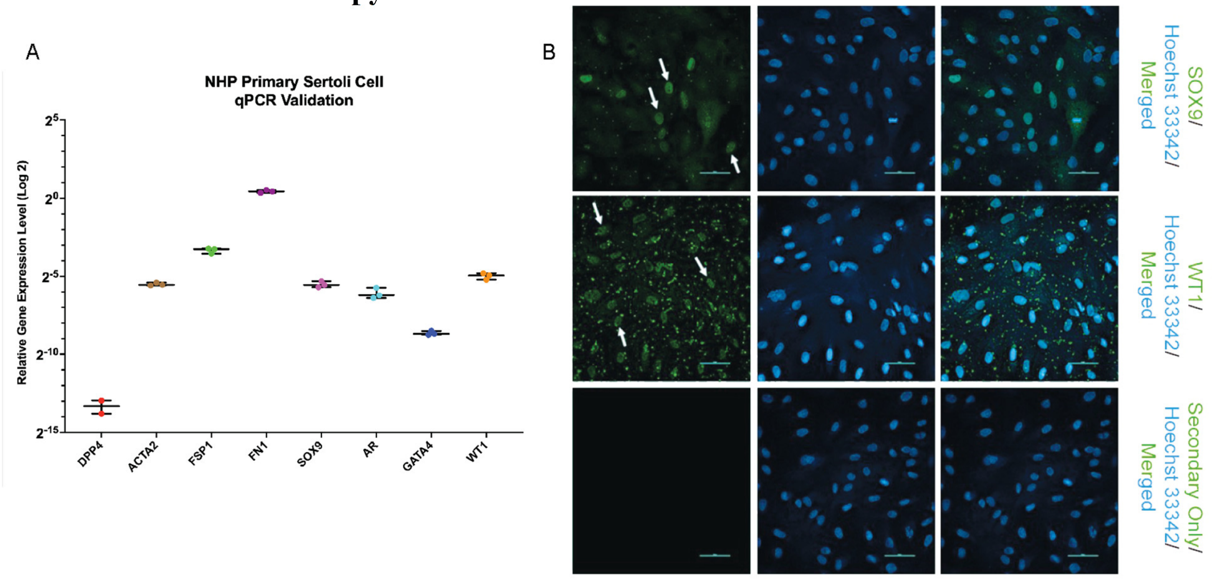

NHP Sertoli cells were validated in vitro using qPCR and fluorescence microscopy. (**A**) Graphical representation of individual relative gene expression levels in the NHP Sertoli cells: Fibroblast markers (*DPP4*, *FSP1*, and *FN1*), Sertoli cell markers (Androgen Receptor: *AR*, *SOX9*, *WT1*, and *GATA4*), Myoid marker (*ACTA2*), spermatogenic markers (*MAGEA4* and *TNP1*), and Leydig cell markers (*STAR* and *INSL3*). Each cell represents the mean of the normalized expression compared to the vehicle control from three (n = 3) replicates per drug per concentration (ΔCT). Data represent the minimum and maximum values. (**B**) Immunofluorescent staining reveals expression of characteristic Sertoli cell markers, revealing expected nuclear staining for *SOX9* (green, **Top Row**), *WT1* (green, **Middle row**), and Hoechst 33342 (blue) dye. White arrows point to positively stained cells. The secondary only control (**Bottom row**) is negative for green fluorescence. Scale bar 50 µm.

Supplemental Figures: Ibuprofen and Naproxen Exposure alter the Blood-testis Barrier in a Novel in vitro model

Figure S5. Single-cell RNA-seq data of male gonad.

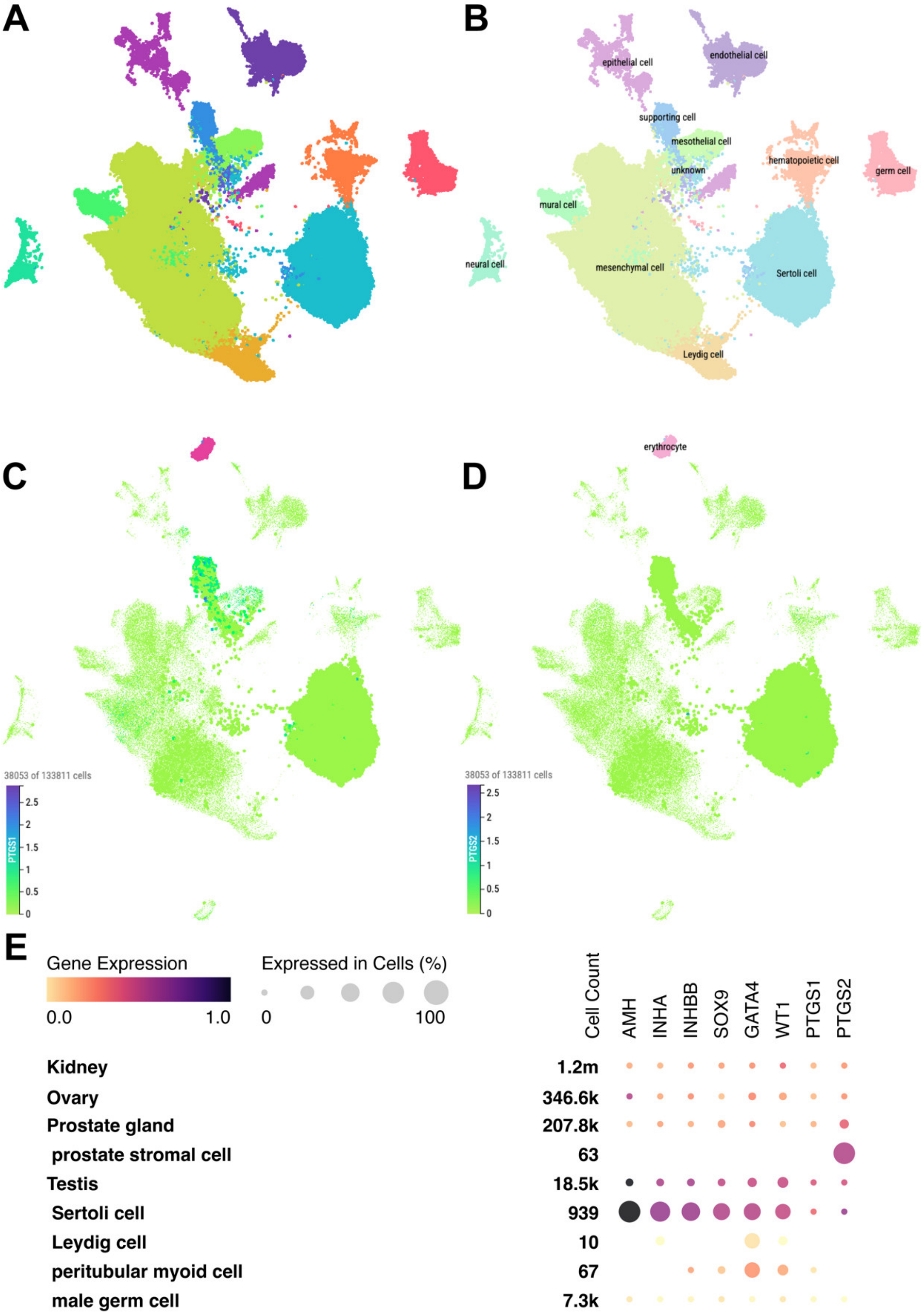

Supplemental Figures: Ibuprofen and Naproxen Exposure alter the Blood-testis Barrier in a Novel in vitro model

Single-cell RNA-seq data of male gonad. (A) UMAP of male gonad samples. (B) Annotated cell populations. (C) *PTGS1* expression in Sertoli cells and supporting cells. (D) *PTGS2* expression in Sertoli cells and supporting cells. (E) Gene expression of Sertoli cell markers, *PTGS1,* and *PTGS2* in kidney, ovary, prostate, and testis tissue shows a small proportion but strong expression of PTGS1 and PTGS2 in Sertoli cells.
