# Supplementary material for: Naproxen and Ibuprofen Exposure Alter the Blood–Testis Barrier in a Novel In Vitro Model
Source: Int J Mol Sci. 2026 Mar 26;27(7):3033. doi: 10.3390/ijms27073033 (PMC13072845; doi:10.3390/ijms27073033)
Supplement: Supplementary file 1 [file ijms-27-03033-s001.zip › 20260226_Supplemental Tables.pdf]

Supplemental Tables: Ibuprofen and Naproxen Exposure alter the Blood-testis Barrier in a Novel in vitro model

Table S1. Oligonucleotide primer sequences.

Gene	Forward Primer Sequence (5'-3')	Reverse Primer Sequence (5'-3')
PTGS1	AACCTTATCCCCAGTCCCCC	CAACTGCTTCTTCCCTTTGGTT
PTGS2	AGGCTTCCATTGACCAGAGC	GCAGACATTTCCTTTTCTCCTGT
AR	GGATGGGGCTCATGGTGTTT	GCCCATCCACTGGAATAATGC
SOX9	GGGCAAGCTCTGGAGACTTCTG	GGGAGATGTGCGTCTGCTC
GATA4	CGGCACCCCAATCTCGTAG	GCGGGAGGCAGACAGC
CLDN3	CGAGTCGTACACCTTGCACT	GGCAGCAACATCATCACGTC
CLDN4	AAAGTGCCTTTGTTGGCCTG	CGAGTGTGAGCAGACCAGTT
CLDN8	GCACTCGTGCTCATTGTTGG	GATCTGGAGTAGACGCTCGG
CLDN11	CACCAATGACTGGGTGGTGA	CAGGACTGAGGCAGCAATCA
NFE2L2	AACTACTCCCAGGTTGCCCA	ATGTGGCCGGGAATATCAGG
DPP4	TGGTCTCCAAACGGCACTTT	TGCCCATGTCACATCACACA
ACTA2	AGCGTGGCTATTCCTTCGTT	TGAAGGATGGCTGGAACAGG
FSP1	CAGAACTAAAGGAGCTGCTGACC	CTTGGAAGTCCACCTCGTTGTC
FN1	CCCATCAGCAGGAACACCTT	GTGGGAGCATCCAGTTTGGT
STAR	GAGACCCAGCAGGACAATGG	ATTAGGGTTCCACTCCCCCA
INSL3	GATATGCCTGATAAGTTGGTCGG	ATGTCGTCTCTCCAGCCACT
WT1	TGAGACCAGTGAGAAACGCC	ATGAGTCCTGGTGTGGGTCT
MAGEA4	GAGGCAAGGTTTTCAGGGGA	GCAGCCTCCTTCTCCTCAGT
TNP1	TGACAGCACAATAGAGCCCC	GGCTGGTCGACATGGTAAGT
GAPDH	ACAACTTTGGTATCGTGGAAGG	GCCATCACGCCACAGTTTC

Oligonucleotide primers used for PCR amplification of cDNAs isolated from vehicle control or NSAID treated non-human primate (NHP) primary Sertoli cells.

Supplemental Tables: Ibuprofen and Naproxen Exposure alter the Blood-testis Barrier in a Novel in vitro model

Table S2. Antibodies used for immunocytochemistry and immunohistochemistry.

Antibody	Company	Catalog Number
Recombinant Anti-COX-1/ COX1(Rabbit)	Abcam	ab109025
PTGS2	Abcam	ab23672
Anti-SOX9 (Rabbit)	EMD Millipore	AB5535
Human SOX9 (Goat)	R&D Systems	AF3075
Recombinant Anti-Wilms Tumor Protein antibody (Rabbit)	Abcam	ab89901
UTF1 Mouse anti-Human	Millipore/ Fisher Scientific	MAB4337MI
Rabbit IgG	BD Pharmingen	550875
Goat IgG	R&D Systems	AB-108-C
Mouse IgG	BD Pharmingen	557273
Goat anti-rabbit Alexa Fluor™ 488	Invitrogen	A11034
Alexa Fluor™ Donkey anti-rabbit 568	Invitrogen	A10042
Alexa Fluor™ Donkey anti-mouse 488	Invitrogen	A21202
Donkey anti-goat Alexa Fluor™ 647	Invitrogen	A21447
Purified Mouse Anti-Cox2	BD Transduction Laboratories™	610203

Supplemental Tables: Ibuprofen and Naproxen Exposure alter the Blood-testis Barrier in a Novel in vitro model

Table S3. Clinical patient data.

	Number of Patients	Average Age of Patients	Diagnosis	Hormone Levels	Cryptorchidism	Varicocele
**Obstructive Azoospermia**	1	34	Full Spermatogenesis	FSH, LH, Testosterone are normal	No	No
**Non-Inflammatory**	4	39.5	Hypospermatogenesis	FSH, LH, Testosterone are normal	No	No
			Maturation Arrest	FSH, LH are normal, Testosterone is low	No	No
			SCOS	FSH (High), LH (High), Testosterone is normal	No	No
			Mixed Atrophy	FSH (High), LH, Testosterone are normal	No	No
**Testicular Inflammation**	8	39	Tubular fibrosis, SCOS	FSH (High), LH (High), Testosterone is normal	No	No
			Tubular fibrosis, SCOS, Mixed atrophy	FSH (High), LH (High), Testosterone is low	Yes	No
			Maturation arrest, mixed atrophy	FSH (High), LH, Testosterone are normal	No	No
			Maturation arrest, mixed atrophy	FSH (High), LH (High), Testosterone is normal	Yes	No
			Mixed atrophy	FSH (High), LH, Testosterone are normal	No	No
			Maturation arrest, mixed atrophy	FSH (High), LH, Testosterone are normal	No	No
			Maturation arrest, mixed atrophy	FSH (High), LH, Testosterone are normal	No	Yes
			Mixed atrophy	FSH (High), LH, Testosterone are normal	Yes	No
**GCNIS**	3	39.33	Mixed atrophy, GCNIS	FSH is normal, LH, Testosterone are unknown	No	No
			Mixed atrophy, tubular fibrosis, GCNIS	FSH (High), LH (High), Testosterone is normal	Yes	No
			GCNIS bilateral, Mixed atrophy	FSH, LH are normal, Testosterone is low	No	No

Supplemental Tables: Ibuprofen and Naproxen Exposure alter the Blood-testis Barrier in a Novel in vitro model

Sixteen male patients consulted an andrologist for male infertility at the Clinic of Urology at the University of Zagreb School of Medicine, University Centre Zagreb, and were subjected to an open biopsy of the testis. Based on their clinical presentation and histology analysis, patients were classified as obstructive azoospermia with a diagnosis of full spermatogenesis or various degrees of testicular parenchyma damage.
