# Supplementary material for: Naproxen and Ibuprofen Exposure Alter the Blood–Testis Barrier in a Novel In Vitro Model
Source: Int J Mol Sci. 2026 Mar 26;27(7):3033. doi: 10.3390/ijms27073033 (PMC13072845; doi:10.3390/ijms27073033)
Supplement: Supplementary file 1 [file ijms-27-03033-s001.zip › 20260226_Supplemental_Table S4.pdf]

Tissue	Cell type	Cell Count	Tissue composition	Gene Symbol	Expression	Expression, Scaled	Number of Cells Expressing Genes
**testis**	Sertoli cell	939	3.82%	*AMH*	3.655072401	1	708
**testis**	Sertoli cell	939	3.04%	*INHA*	2.75702132	0.599192604	563
**testis**	Sertoli cell	939	2.64%	*INHBB*	2.721668454	0.583414337	489
**testis**	Sertoli cell	939	2.48%	*SOX9*	2.599574877	0.528922995	460
**testis**	Sertoli cell	939	2.27%	*GATA4*	2.575472586	0.51816595	420
**testis**	Sertoli cell	939	1.93%	*WT1*	2.493250743	0.481469682	357
**testis**	Sertoli cell	939	0.03%	*PTGS1*	2.211897373	0.355899418	6
**testis**	Sertoli cell	939	0.02%	*PTGS2*	2.741604487	0.592311948	3
**testis**	Leydig cell	10		*AMH*			
**testis**	Leydig cell	10	0.01%	*INHA*	1.41470325	0.000105291	1
**testis**	Leydig cell	10		*INHBB*			
**testis**	Leydig cell	10		*SOX9*			
**testis**	Leydig cell	10	0.02%	*GATA4*	1.562341213	0.065997297	4
**testis**	Leydig cell	10	0.01%	*WT1*	1.414467335	0	1
**testis**	Leydig cell	10		*PTGS1*			
**testis**	Leydig cell	10		*PTGS2*			
**testis**	peritubular myoid cell	67		*AMH*			
**testis**	peritubular myoid cell	67		*INHA*			
**testis**	peritubular myoid cell	67	0.01%	*INHBB*	1.881940365	0.20863696	2
**testis**	peritubular myoid cell	67	0.02%	*SOX9*	1.709469636	0.131661892	3
**testis**	peritubular myoid cell	67	0.14%	*GATA4*	1.96632708	0.246299428	26
**testis**	peritubular myoid cell	67	0.05%	*WT1*	1.858294805	0.198083757	9
**testis**	peritubular myoid cell	67	0.01%	*PTGS1*	1.54793191	0.059566309	2
**testis**	peritubular myoid cell	67		*PTGS2*			
**testis**	male germ cell	7259	0.01%	*AMH*	1.533930421	0.053317333	1
**testis**	male germ cell	7259	0.02%	*INHA*	1.442604303	0.012557755	4
**testis**	male germ cell	7259	0.01%	*INHBB*	1.469240904	0.024445883	2
**testis**	male germ cell	7259	0.05%	*SOX9*	1.547776752	0.059497061	9

testis	male germ cell	7259	0.07%	*GATA4*	1.455668376	0.018388355	13
testis	male germ cell	7259	0.03%	*WT1*	1.540590668	0.056289854	5
testis	male germ cell	7259	0.02%	*PTGS1*	1.442879915	0.012680762	4
testis	male germ cell	7259	0.01%	*PTGS2*	1.420627475	0.00274932	1
prostate gland	aggregated	207838	0.45%	*PTGS1*	1.821721373	0.181760742	940
prostate gland	aggregated	207838	10.95%	*PTGS2*	2.257242206	0.37613718	22761
kidney	aggregated	1245614	1.07%	*PTGS1*	1.786740447	0.166148474	13341
kidney	aggregated	1245614	0.70%	*PTGS2*	1.958766386	0.242925029	8682
liver	aggregated	1444734	0.71%	*PTGS1*	1.823138375	0.182393161	10216
liver	aggregated	1444734	2.05%	*PTGS2*	2.344738208	0.415187347	29686
ovary	aggregated	346569	1.48%	*PTGS1*	1.77660475	0.161624831	5122
ovary	aggregated	346569	1.59%	*PTGS2*	1.977742565	0.25139425	5509

Table S4. Single-cell gene expression data of Sertoli cell-specific markers and *PTGS1* (*COX1*) and *PTGS2* (*COX2*) in Sertoli cell, Leydig cell, peritubular myoid cell, male germ cells[1, 2]. *PTGS1* and *PTGS2* expressions from the prostate gland, kidney, liver, and ovary are shown on the bottom[1, 2]. Although a small portion, high expression of *PTGS1* and *PTGS2* was found in Sertoli cells.

[1] C.S.-C.B. Program, S. Abdulla, B. Aevermann, P. Assis, S. Badajoz, S.M. Bell, E. Bezzi, B. Cakir, J. Chaffer, S. Chambers, J. Michael Cherry, T. Chi, J. Chien, L. Dorman, P. Garcia-Nieto, N. Gloria, M. Hastie, D. Hegeman, J. Hilton, T. Huang, A. Infeld, A.-M. Istrate, I. Jelic, K. Katsuya, Y.J. Kim, K. Liang, M. Lin, M. Lombardo, B. Marshall, B. Martin, F. McDade, C. Megill, N. Patel, A. Predeus, B. Raymor, B. Robatmili, D. Rogers, E. Rutherford, D. Sadgat, A. Shin, C. Small, T. Smith, P. Sridharan, A. Tarashansky, N. Tavares, H. Thomas, A. Tolopko, M. Urisko, J. Yan, G. Yeretssian, J. Zamanian, A. Mani, J. Cool, A. Carr, CZ CELL×GENE Discover: A single-cell data platform for scalable exploration, analysis and modeling of aggregated data, bioRxiv (2023) 2023.10.30.563174.
[2] L. Garcia-Alonso, V. Lorenzi, C.I. Mazzeo, J.P. Alves-Lopes, K. Roberts, C. Sancho-Serra, J. Engelbert, M. Mareckova, W.H. Gruhn, R.A. Botting, T. Li, B. Crespo, S. van Dongen, V.Y. Kiselev, E. Prigmore, M. Herbert, A. Moffett, A. Chedotal, O.A. Bayraktar, A. Surani, M. Haniffa, R. Vento-Tormo, Single-cell roadmap of human gonadal development, Nature 607(7919) (2022) 540-547.
