# Supplementary figures and images for: Naproxen and Ibuprofen Exposure Alter the Blood–Testis Barrier in a Novel In Vitro Model
Source: Int J Mol Sci. 2026 Mar 26;27(7):3033. doi: 10.3390/ijms27073033 (PMC13072845; doi:10.3390/ijms27073033)

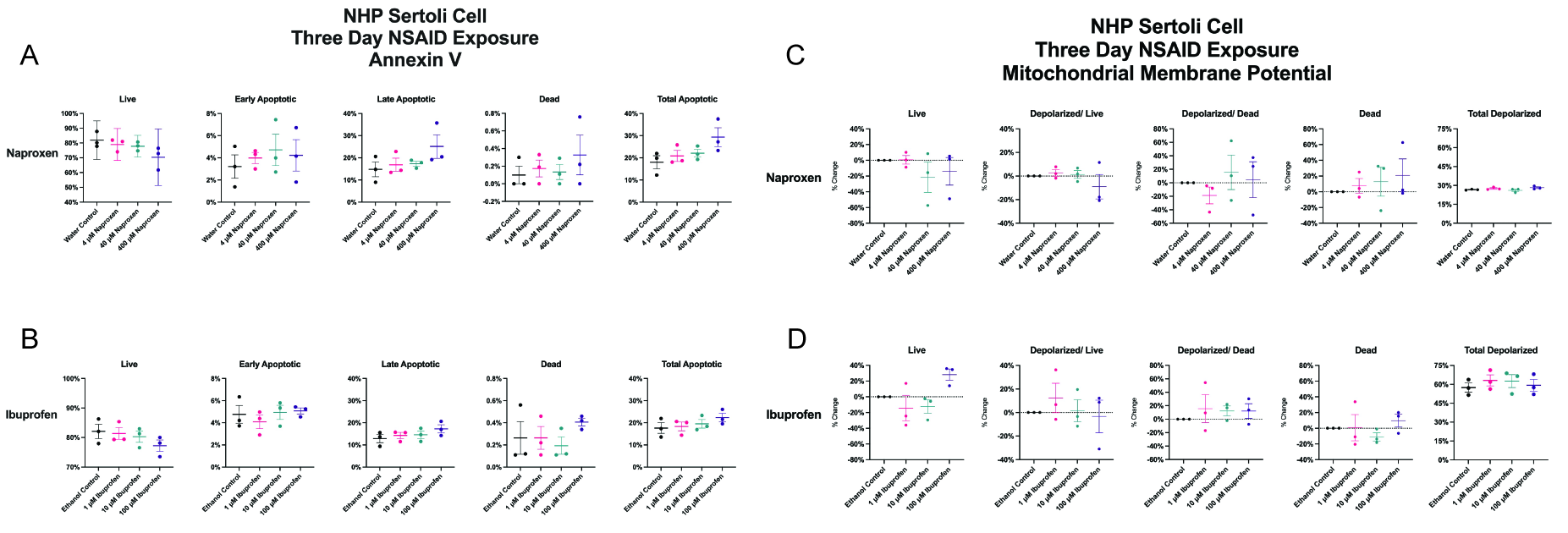

Supplement: Supplementary file 1 [file ijms-27-03033-s001.zip › Figure S1.tif]

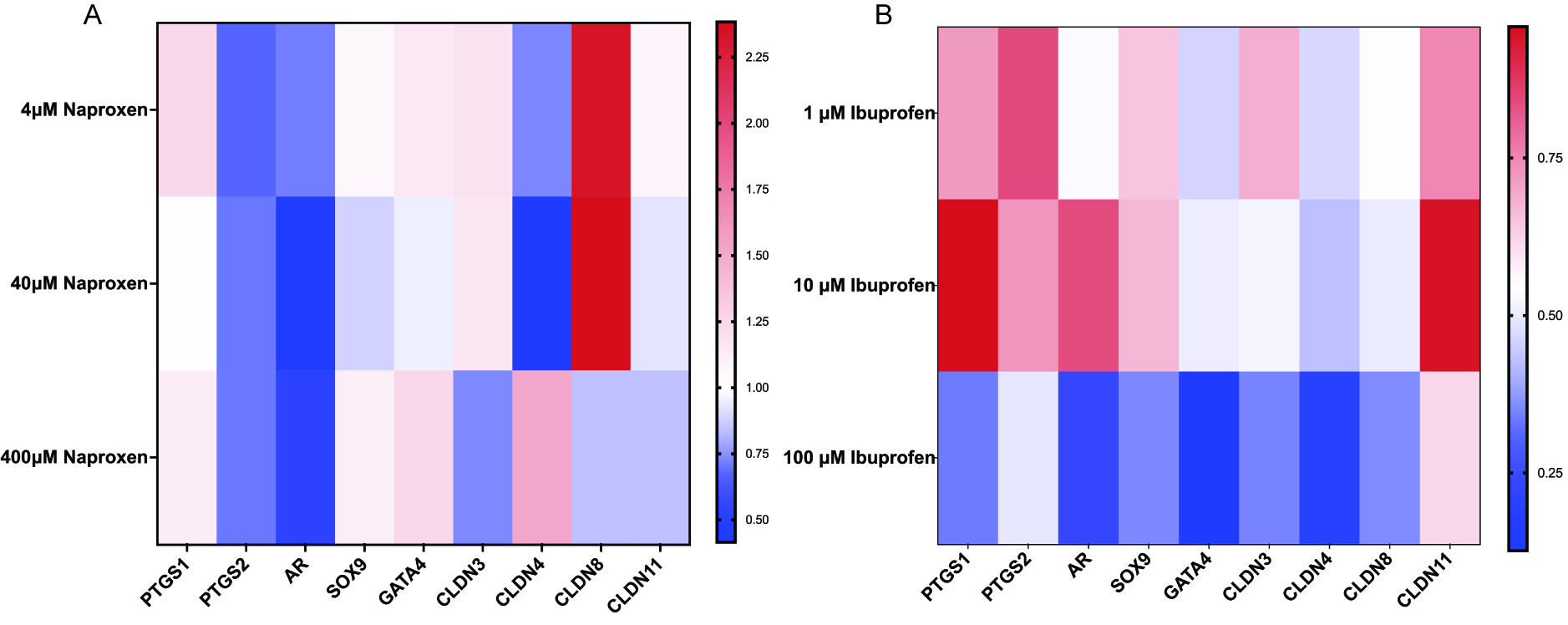

Supplement: Supplementary file 1 [file ijms-27-03033-s001.zip › Figure S2.tif]

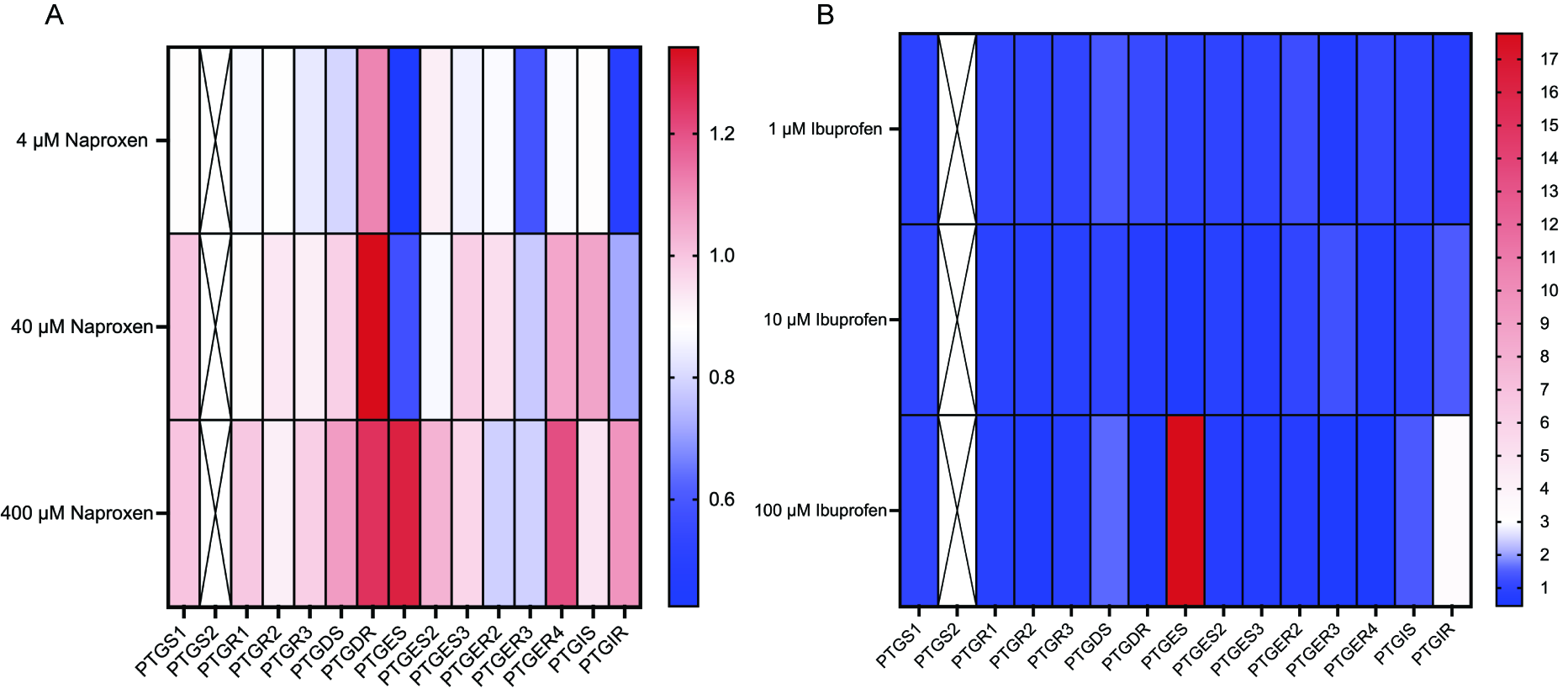

Supplement: Supplementary file 1 [file ijms-27-03033-s001.zip › Figure S3.tif]

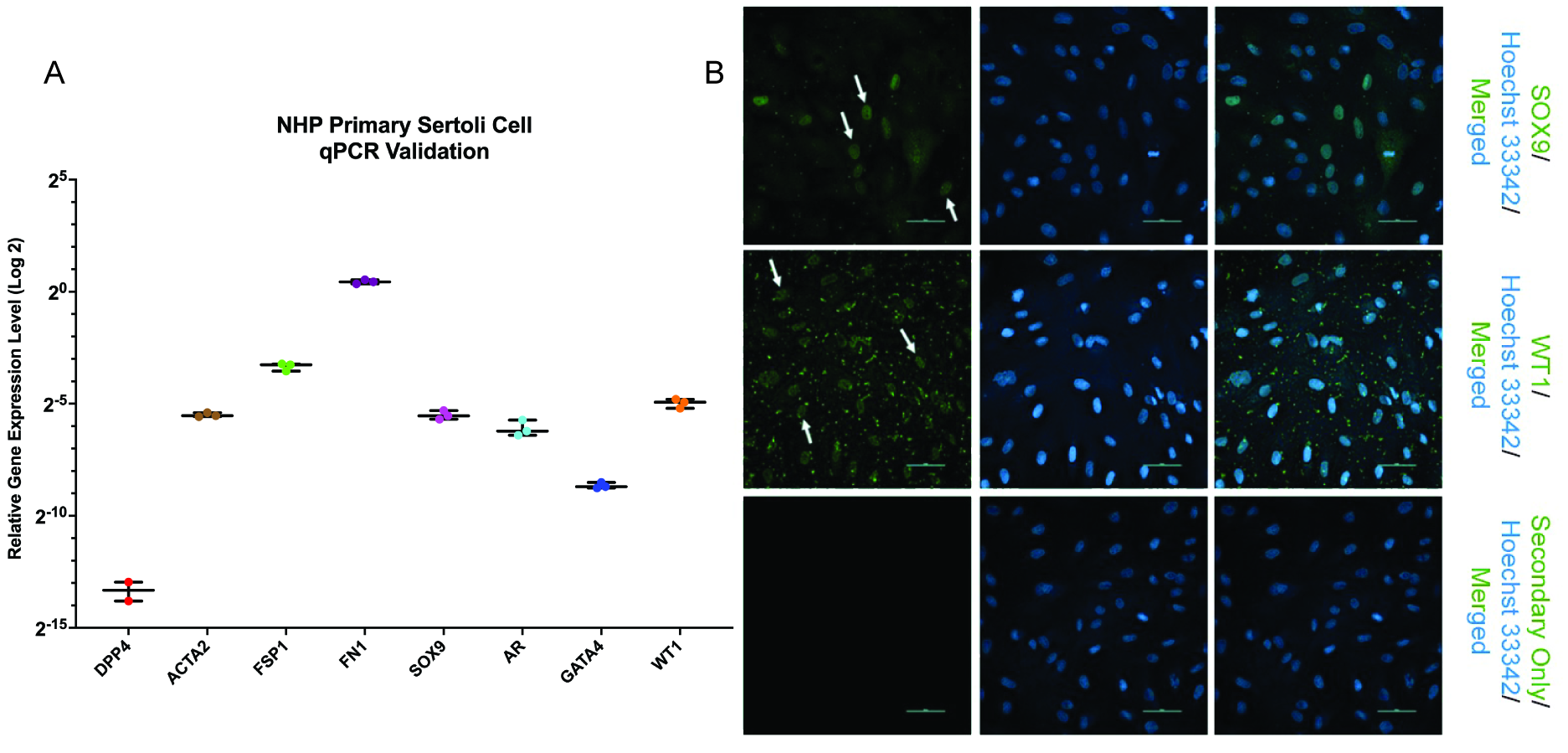

Supplement: Supplementary file 1 [file ijms-27-03033-s001.zip › Figure S4.tif]

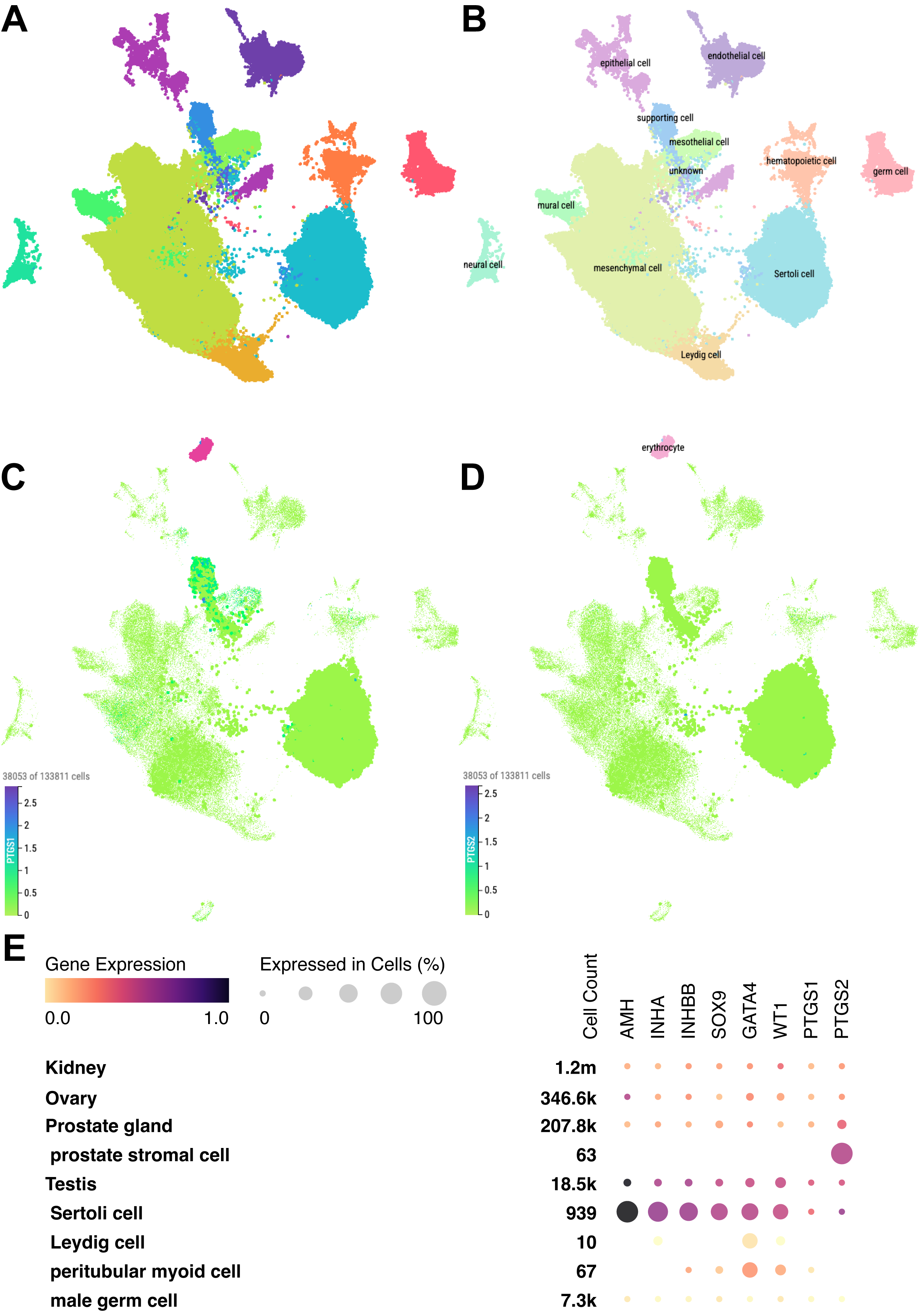

Supplement: Supplementary file 1 [file ijms-27-03033-s001.zip › Figure S5.tif]
